# Maintenance of Taste Organs Is Strictly Dependent on Epithelial Hedgehog/GLI Signaling

**DOI:** 10.1371/journal.pgen.1006442

**Published:** 2016-11-28

**Authors:** Alexandre N. Ermilov, Archana Kumari, Libo Li, Ariell M. Joiner, Marina A. Grachtchouk, Benjamin L. Allen, Andrzej A. Dlugosz, Charlotte M. Mistretta

**Affiliations:** 1 Department of Dermatology, Medical School, University of Michigan, Ann Arbor, Michigan, United States of America; 2 Department of Biologic and Materials Sciences, School of Dentistry, University of Michigan, Ann Arbor, Michigan, United States of America; 3 Department of Cell and Developmental Biology, Medical School, University of Michigan, Ann Arbor, Michigan, United States of America; Stanford University School of Medicine, UNITED STATES

## Abstract

For homeostasis, lingual taste papilla organs require regulation of epithelial cell survival and renewal, with sustained innervation and stromal interactions. To investigate a role for Hedgehog/GLI signaling in adult taste organs we used a panel of conditional mouse models to manipulate GLI activity within epithelial cells of the fungiform and circumvallate papillae. Hedgehog signaling suppression rapidly led to taste bud loss, papilla disruption, and decreased proliferation in domains of papilla epithelium that contribute to taste cells. Hedgehog responding cells were eliminated from the epithelium but retained in the papilla stromal core. Despite papilla disruption and loss of taste buds that are a major source of Hedgehog ligand, innervation to taste papillae was maintained, and not misdirected, even after prolonged GLI blockade. Further, vimentin-positive fibroblasts remained in the papilla core. However, retained innervation and stromal cells were not sufficient to maintain taste bud cells in the context of compromised epithelial Hedgehog signaling. Importantly taste organ disruption after GLI blockade was reversible in papillae that retained some taste bud cell remnants where reactivation of Hedgehog signaling led to regeneration of papilla epithelium and taste buds. Therefore, taste bud progenitors were either retained during epithelial GLI blockade or readily repopulated during recovery, and were poised to regenerate taste buds once Hedgehog signaling was restored, with innervation and papilla connective tissue elements in place. Our data argue that Hedgehog signaling is essential for adult tongue tissue maintenance and that taste papilla epithelial cells represent the key targets for physiologic Hedgehog-dependent regulation of taste organ homeostasis. Because disruption of GLI transcriptional activity in taste papilla epithelium is sufficient to drive taste organ loss, similar to pharmacologic Hedgehog pathway inhibition, the findings suggest that taste alterations in cancer patients using systemic Hedgehog pathway inhibitors result principally from interruption of signaling activity in taste papillae.

## Author Summary

Taste papillae are small organs visible on the surface of the tongue that contain taste buds, which are connected to nerves that transmit signals for taste sensation to the brain. To function properly, taste papilla and taste bud cells need to be continuously replenished. We are studying how collections of proteins, called signaling pathways, ensure that the sense of taste is maintained. We show that one specific signaling pathway, the Hedgehog pathway, is absolutely essential for proper function of taste organs. When we block the Hedgehog pathway, nearly all taste papillae dramatically change shape and taste buds disappear. This response occurs because specific taste cell populations in the taste papillae can no longer function properly, even though taste organ nerves are still present. When we release the blockade of Hedgehog signaling, many taste papillae and taste buds are regenerated. Our findings identify a critical requirement for the Hedgehog signaling pathway in maintaining taste papillae and taste buds, help explain why cancer patients treated with Hedgehog pathway inhibitors lose their ability to taste, and suggest that changes in this pathway could be responsible for other conditions associated with taste disturbance.

## Introduction

Hedgehog (HH) signaling plays complex regulatory roles in adult organ and tissue maintenance [1]. From regulation in epithelia that turn over slowly and normally are ‘quiescent’ [2] to skin that regularly renews [3], roles for HH activity are temporally- and niche-specific, and rely on interactions with nerves [4] and stromal cells [5,6]. Delineating the context-dependent functions of HH signaling in different tissues is thus a high priority for better understanding the normal regulation of organ homeostasis, regeneration and disease. Taste papillae are constantly renewing, complex, multimodal sensory organs that subserve lingual taste, touch and temperature, and have varied and essential roles in eating [7]. The specialized taste bud cells turn over every 3 to 20-plus days, with an average life span of about 10 days [8–11]. The stratified squamous epithelium of the papilla organs also continuously turns over [12,13] and is seated on a basal lamina that envelopes a connective tissue core of stromal fibroblasts, blood vessel endothelial cells, nerve fibers and ensheathing Schwann cells, and extracellular matrix. Despite constant taste bud and epithelial cell renewal and replacement, and dynamic connective tissues, the lingual taste organs maintain structural and functional sensory integrity. The precise regulation that orchestrates the biology of such diverse cell types to sustain taste papilla organs and lingual sensory homeostasis is not well understood. We have approached study of taste organ maintenance and renewal with multiple genetic mouse models to focus on regulation by Hedgehog/GLI (HH/GLI) signaling.

Hedgehog (HH) signaling initiates when secreted HH ligands bind to Patched1 (PTCH1) and to the co-receptors GAS1, CDON and BOC [14], resulting in de-repression of Smoothened (SMO) which transduces the HH signal intracellularly via a series of cytoplasmic intermediaries [15,16]. Subsequent modulation of the GLI transcription factors (GLI1, GLI2, GLI3) leads to differential protein processing, including a shift from transcriptional repressor to activator forms, and transcription of HH target genes that include *Ptch1* and *Gli1* [17]. GLI1 functions strictly as a transcriptional activator but is dispensable for embryonic and postnatal development [18,19]. GLI2 is the major activator of HH-driven transcriptional responses *in vivo* [20], whereas GLI3 operates primarily as a repressor [21].

The HH pathway is a principal regulator of taste organ development although other pathways are involved [22,23]. There are well characterized effects of the HH pathway in taste papilla induction and patterning, and proposed roles in taste bud progenitor differentiation [24– 26]. Significantly, recent studies point to a requirement for properly regulated HH signaling in adult taste organ maintenance. Expression of an oncogenic form of GLI2 in tongue epithelia leads to altered fungiform papilla number, structure, taste buds, and epithelial proliferation [27]. In contrast, overexpression of SHH results in the formation of ectopic, non-innervated taste bud-like structures in suprabasal lingual epithelium outside of the fungiform papillae [28]. In cancer patients, treatment with drugs to inhibit the HH pathway is associated with profound alterations in taste [29,30], and use of these drugs to block HH signaling in rodents results in aberrant fungiform papillae, loss of taste buds and severely disrupted taste sensation [31].

Fungiform papilla (FP) epithelia contain stem or progenitor cells that replenish taste bud cells during tissue homeostasis [8, 32,33], and with genetic-inducible fate mapping, we showed that *Gli1*-positive, HH-responding cells in the basal layer of fungiform papillae and in perigemmal cells contribute both to taste bud cells and to taste papilla epithelium [27]. Putative stem cells also have been reported within taste buds themselves [34], and SHH-positive progenitor cells within taste buds can give rise to all taste bud cell types [35]. Additionally, forced activation of Wnt signaling in SHH-expressing cells promotes FP taste cell differentiation [36].

In the circumvallate papilla (CV) on the posterior tongue, also, taste and papilla cells are in constant turnover and Lgr5 is a proposed stem cell marker [34]. HH pathway regulation has been suggested in taste bud cell proliferation and differentiation [37]. SHH is detected in taste bud basal cells and *Ptch1*-expressing cells are included in highly proliferative epithelial cells around taste buds, but reportedly not in the underlying stroma [9,38].

The patterns in FP and CV suggest a paracrine mode of signaling from SHH-expressing taste bud cells to neighboring epithelial and stromal cell populations [27,37]. However, to date, a requirement for HH signaling activity in specific cell populations of adult taste organs has not been rigorously examined with genetic HH pathway blockade.

To gain a better understanding of how HH signaling contributes to taste organ maintenance, we genetically manipulated GLI transcription factor activity selectively in epithelial cells using either a doxycycline-regulated dominant-negative *Gli* repressor, *GliR*, allele, or by conditional deletion of epithelial *Gli2* either on a wild-type or *Gli1* null background. We have characterized rapid and profound effects of epithelium-specific HH/GLI blockade on FP and CV papilla integrity and taste bud loss, and a relative sparing of neural and stromal elements. Strikingly, the FP, CV and taste bud phenotypes were rescued when GLI inhibition was stopped, demonstrating a remarkable plasticity in these tissues; although in a subset of fungiform papillae there was no recovery. Overall we show that HH signaling is an essential regulator of papilla taste organ integrity, principally with epithelial effects that respond rapidly both to signal repression and release. Our findings argue that taste alterations in patients treated with systemic HH pathway inhibitors can be explained by interruption of HH/GLI signaling activity in HH-responsive epithelial cells of taste papilla organs.

## Results

### Blocking GLI function in epithelial cells leads to fungiform papilla disruption and taste bud loss

#### Blockade of Gli activity using a dominant-negative GLI repressor

**Fig 1:**
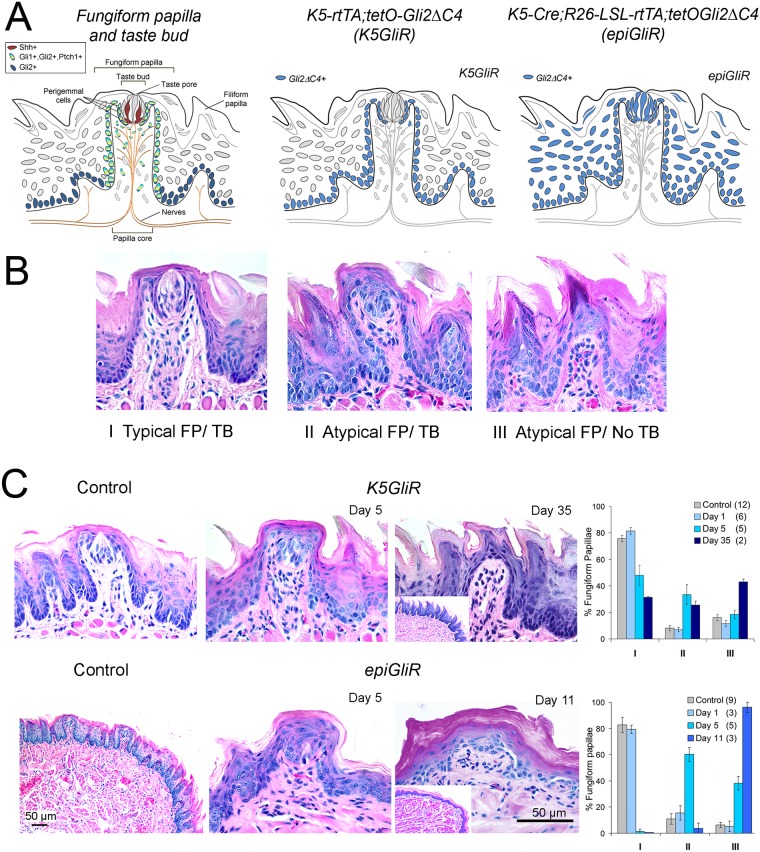
Inhibition of epithelial HH/GLI signaling leads to alterations in fungiform papillae and epithelium and loss of taste buds. **A**. Fungiform papilla and taste bud diagrams to illustrate cells types and location of HH pathway signaling elements. Canonical HH signaling is restricted to cells expressing both *Gli1* and *Ptch1* (Control Papilla and Taste bud, left). Center and right panel diagrams illustrate cell/tissue areas (blue) of dominant-negative transgene expression in doxycycline-inducible *K5GliR* and *epiGliR* mouse models. **B**. Three types of Fungiform Papilla and Taste Bud (FP/TB) used for quantifying effects of HH repression: I, Typical FP/TB: Typical papilla morphology with intact apical taste bud; II, Atypical FP/TB: Atypical, mis-shapen papilla morphology and taste bud remnant or cluster of distinctly staining cells; III, Atypical FP/No TB: Atypical papilla morphology with keratinized apical point and no discernable taste bud cells. **C**. H&E sections illustrate FP/TB morphology for Control and *K5GliR* and *epiGliR* mouse tongues at indicated time points after transgene induction. Scale bar applies to all micrographs except low power and insets. Low power image for *epiGliR* Control illustrates overall tongue morphology. Inset at Day 11 indicates disruption of fungiform and filiform papillae but at Day 35 *K5GliR*, inset illustrates intact filiform. Graphs present quantification of TYPE I, II and III taste organs at increasing durations of transgene activation. TYPE I (Typical) FP/TB are substantially reduced (*K5GliR*) or eliminated (*epiGliR*) whereas TYPE III (Atypical/No TB) organs accumulate. Numbers of tongues at each time point are included in parentheses in graph legends. Full statistics for ANOVA are presented in S1A and S1B Fig.

C-terminal deletion of the GLI2 transactivation domain yields proteins with potent GLI dominant-negative activity [39,40], and expression of a *GLI2ΔC4* mutant in mouse skin epithelia mimics the phenotype seen with epithelial deletion of *Smo* to block HH signaling [41,42]. To conditionally block HH-driven transcriptional responses in tongue we produced transgenic mice carrying the *Gli2ΔC4* mutant downstream of *tetO* response elements, and generated either *K5-rtTA;tetO-Gli2ΔC4 (K5GliR)* or *K5-Cre;R26-lsl-rtTA;tetO-Gli2ΔC4 (epiGliR)* mice. In doxycycline-treated *K5GliR* mice, GLI activity is blocked by *GLI2ΔC4* in K5-expressing basal cells; whereas in *epiGliR* mice, *Gli2ΔC4* is expressed and GLI activity blocked throughout the lingual epithelium (Fig 1A).

To characterize the phenotypic response of taste organs to HH/GLI blockade, we classified fungiform papillae (FP) and taste buds (TB) as TYPE I (Typical FP and Typical TB), TYPE II (Atypical FP and Atypical TB), or TYPE III (Atypical FP and No TB) (Fig 1B), and we quantified the proportion of fungiform papilla/taste bud phenotypes at multiple time points after GLI blockade. No phenotypic alterations were detected in either *K5GliR* or *epiGliR* mice 1 day after starting doxycycline treatment (Fig 1C). In contrast, after 5 days of transgene activation in *K5GliR* mice only about 50% of taste organs were typical (TYPE I); after 35 days, the proportion of typical taste organs was reduced to about 35%. Notably, by 35 days about 40% of taste organs had no detectable taste bud (Fig 1C *K5GliR*, TYPE III). In *epiGliR* mice, with GLI function blocked in all epithelial cell compartments of the tongue, the effect was faster and more robust. After 5 days very few typical taste organs (TYPE I) remained; by 11 days almost all taste organs had no detectable taste bud (TYPE III) (Fig 1C *epiGliR*). In both models the remaining aberrant fungiform papillae had acquired multiple layers of cornified cells at the papilla apex, and in *epiGliR mice*, many fungiform papillae did not retain the deep epithelial down-growths into the stroma that characterize their typical rectangular papilla shape (Fig 1C, Day 11). (Complete ANOVA data and posthoc analyses are provided in S1A and S1B Fig)

#### Gli2 gene deletion

**Fig 2:**
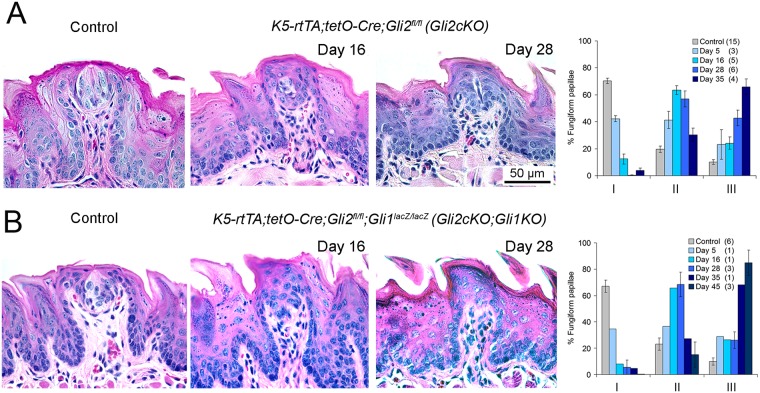
Conditional deletion of Gli2 in tongue epithelia leads to loss of Typical fungiform papillae and taste buds and accumulation of Atypical taste organs. H&E sections illustrate loss of Typical FP/TB morphology after 16 and 28 days of epithelium-targeted deletion of *Gli2* in *Gli2cKO* (A) and *Gli2cKO;Gli1KO* (B) mouse tongues, compared to Control. Scale bar in A refers to all images. Graphs present quantification of TYPE I, II and III taste organs at increasing durations of gene deletion, demonstrating essential elimination of TYPE 1, Typical FP/TB after about one month with progressive accumulation of TYPE III FP/No TB. Numbers of tongues at each time point are included in parentheses in graph legends. Full statistics for ANOVA are presented in S1C and S1D Fig.

To confirm the requirement for HH/GLI signaling function in FP/TB maintenance, we conditionally deleted *Gli2* in the epidermis of *K5-rtTA;tetO-Cre;Gli2*^*fl/fl*^ mice (referred to as *Gli2cKO*) up to 35 days. In these animals, taste bud cells were gradually lost and FP apices became progressively more heavily cornified (Fig 2A *Gli2cKO*). Comparable to timing in *K5GliR* and *epiGliR* mice, after 5 days of doxycycline there was a significant reduction in Typical FP/TB (TYPE I); after 16 days, TYPE I were reduced to about 15% of all FP and were virtually eliminated after 28 days (Fig 2A). After 35 days, more than 65% of FP had no TB (TYPE III). (ANOVA data and posthoc analyses are included in S1C Fig).

In mice with an epithelial *Gli2-* and global *Gli1*-deletion (*K5-rtTA;tetO-Cre;Gli2*^*fl/fl*^*;Gli1*^*lacZ/lacZ*^, *referred to as Gli2cKO;Gli1KO)* the extent and timing of FP/TB alteration were similar to those in mice with *Gli2* deletion alone (Fig 2B *Gli2cKO;Gli1KO*). These data support the concept that GLI2 is the primary transcriptional activator mediating responses to HH signaling in peripheral taste organs, with little if any contribution from GLI1. Typical FP/TB (TYPE I) were reduced to less than 10% after 16 days of doxycycline administration, and after 35 days more than 60% of FP had no TB (TYPE III) (Fig 2B). Continuing gene induction through 45 days led to virtual elimination of TYPE I FP/TB (Fig 2B). Similar to FP effects in *Gli2cKO* tongues, the apical fungiform papilla epithelium where a taste bud would normally reside acquired a spinous cap with expanded epithelial cell layers (Fig 2B). (ANOVA data and posthoc analyses are provided in S1D Fig).

Overall these data indicate that disruption of GLI transcriptional activity in lingual epithelium using several genetic approaches leads to rapid and significant loss of typical fungiform papillae and taste buds.

### K5-targeted GLI blockade leads to loss of HH-responding cells in fungiform papilla epithelium

**Fig 3:**
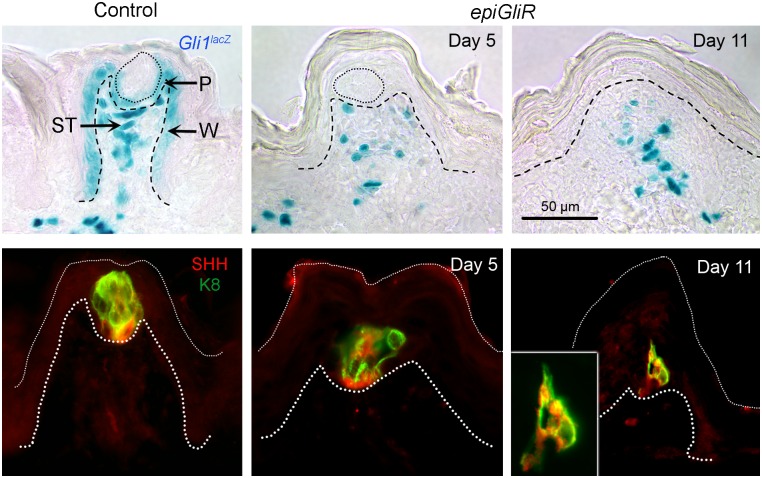
HH-responding cells are lost from the papilla epithelium but retained in connective tissue, and taste bud size, and SHH expression, decrease with duration of HH/GLI repression. **TOP ROW**: X-Gal staining to detect β-gal-positive, HH-responding cells in Control *Gli1*^*lacZ/+*^ mice is typically in perigemmal cells (P), basal epithelial cells of the FP wall (W), and cells of the stromal core (ST). After *epiGliR* transgene activation for 5 days, and continuing at 11 days, there is loss of detectable HH signaling in epithelium with *lacZ*-positive cells in the FP stroma only. **BOTTOM ROW**: SHH is expressed principally within taste buds cells (K8) in Control FP. During HH/GLI repression in *epiGliR* tongues, after 5 days remaining taste buds are progressively reduced in size and SHH ligand is reduced in association with taste cell loss. After 11 days very few TB remnants are observed (see Fig 1 data) and these are very small cell collections. Inset at Day 11 illustrates the nature of the cell remnants that remain in a very small percentage of FP.

To assess effectiveness of signaling blockade in our models we stained for *lacZ* positive cells in mice carrying a *Gli1*^*lacZ*^ allele, which serves as a sensitive and specific reporter for HH pathway activity [18]. In Control mice, HH-responding cells were detected in the basal cell layer of the FP epithelial walls, in perigemmal epithelial cells around the TB, and in stromal cells of the connective tissue core (Fig 3 Control *Gli1*^*lacZ*^). As early as 5 days after transgene activation in *epiGliR* mice, HH-responding cells were no longer detected in the fungiform papilla walls and perigemmal epithelial cells but were retained in the FP connective tissue core (Fig 3, *epiGliR* Day 5, Day 11), confirming repression of HH signaling selectively in the targeted cell populations. Similar results were obtained in *K5GliR*, *Gli2cKO* and *Gli2cKO;Gli1KO* mice (S2A Fig).

### Taste bud cell loss and reduced SHH expression in fungiform papillae

Having established effective blockade of epithelial HH/GLI signaling activity we examined expression of SHH, which in the FP is typically restricted to a subset of taste bud cells [27] that express the taste bud cell marker Keratin 8 (K8) [43]. After only 5 days of transgene expression/GLI blockade in *epiGliR* mice, taste bud cells were fewer in number, did not span the full thickness of the epithelium, and apical taste pores were missing (Fig 3, SHH/K8 Control and Day 5). By 11 days (*epiGliR*) or 35 days (*K5GliR*) of transgene expression, discernible taste bud cell collections were detected only occasionally and they were localized to the lower third of lingual epithelium (Fig 3, Day 11 and inset; S2B Fig, Day 35 *K5GliR*). Overall, within the TB remnants at various stages of “deterioration” associated with HH signaling repression, there were some SHH expressing cells in the TB base as is typical in adult FP/TB (Fig 3, Day 5, Day 11 and inset; S2B Fig, Day 35 *K5GliR*).

The residual SHH in some FPs may be capable of signaling to HH-responsive stromal cells retained in the FP after HH repression and could potentially participate in maintaining epithelial integrity and/or FP structure. There are, however, TYPE III FP/No TB in all models (Figs 1 and 2) and because these have no TBs they have no SHH in the FP.

### Proliferation effects in a sub-set of fungiform papilla epithelial cells

**Fig 4:**
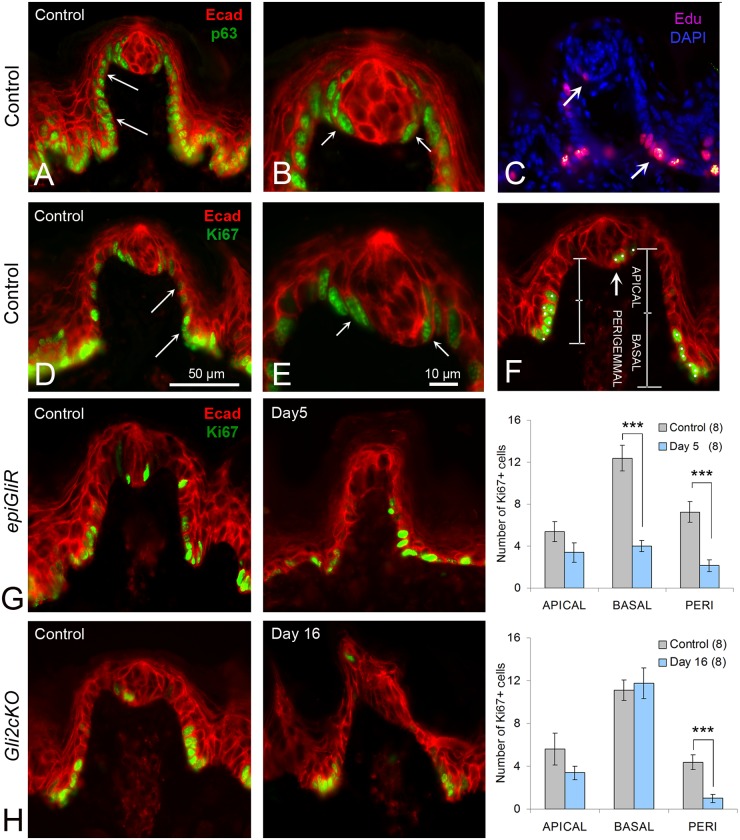
Inhibition of epithelial HH/GLI signaling leads to decreased cell proliferation in specific domains of the fungiform papilla. **A,B**. p63-positive proliferating cells are continuous in basal epithelium of the FP (A, arrows) and in perigemmal cells around the taste bud (B,arrows). **C**. Proliferating cells labeled with EdU are prominent at the base of the FP (arrow) and in the perigemmal region (arrow). **D,E**. Ki67 positive, proliferating cells are numerous in basal epithelial cells at the FP base, are decreasing in number toward FP apical half (D, arrows), and are in the perigemmal region (E, arrows). **F**. Section through a TYPE I, Typical FP/TB with labels to indicate regions for Ki67-positive cell counts in APICAL and BASAL halves of the FP walls and in the PERIGEMMAL region. **G**. Region-specific, proliferation (Ki67+ cells) in Control *epiGliR* mice; reduced proliferation after 5 days transgene activation (Day 5); and graph for quantified data. Bars between pairs in graph indicate statistical significance at p < 0.001 (***). After only 5 days, cell proliferation was reduced in the basal FP wall (BASAL: t = 6.21, p< 0.001) and in the perigemmal region (PERI: t = 4.57, p<0.001). **H**. Cell proliferation in *Gli2cKO* mice in Control; after 16 days of gene deletion (Day 16); and graph for quantified data. Bars between pairs in graph indicate statistical significance at p < 0.001 (***). Cell proliferation was reduced in the perigemmal region (PERI: t = 4.34, p < 0.001). Numbers in parentheses for graphs refer to number of FP analyzed. E- cadherin (Ecad, red) immunoreactions were used to label the epithelium throughout images. Scale bar in D applies to A,C,D,F,G,H. Scale bar in E applies to B,E.

The effects of HH signaling on proliferation in different cell types within tissues are highly context-dependent. Whereas HH signaling drives proliferation in several settings [1], in others the HH pathway maintains quiescence [2]. Altered proliferative activity in papilla organs could contribute to the profound morphological alterations we detected following GLI blockade. Using antibodies to the basal layer marker, p63, the proliferation marker, Ki67, and EdU labeling to detect cells in S phase, we confirmed previously identified domains of high proliferation at the base of the FP and in perigemmal cells (27) in Control tongues (Fig 4A–4E). The marker p63 was expressed uniformly within basal cells of the FP walls and perigemmal cells (Fig 4A; S3A and S3B Fig) whereas the intensity of Ki67 immunostaining was typically reduced in basal cells as they ‘ascended’ the papilla wall toward the apex (Fig 4D).

To quantify proliferation we counted Ki67-positive cells in three epithelial compartments: the *apical* and *basal* halves of the fungiform papillae walls, and the *perigemmal* compartment surrounding the taste bud (Fig 4F). In the *basal* papilla domain of *epiGliR* mice, after 5 days there was a 66% reduction in Ki67-positive cells and a trend toward reduction in the *apical* domain that did not reach significance (Fig 4G APICAL, BASAL). In *Gli2cKO* tongues, after 16 days, there was again a non-significant trend toward fewer Ki67+ cells in the *apical* FP but there was no difference in the *basal* papilla (Fig 4H APICAL, BASAL). The difference in *basal* papilla proliferation in *epiGliR* compared to *Gli2cKO* FP could relate to the more profound effects on papilla structure in the *epiGliR* model (see Fig 1).

Strikingly, GLI blockade led to a reduction in Ki67-positive *perigemmal* FP cells both in *epiGliR* and *Gli2cKO* mice (Fig 4G *epiGliR* PERI; H *Gli2cKO* PERI). This reflects the fact that the number of *perigemmal* cells in the region that neighbors taste bud ‘remnants’ is radically reduced in GLI-inhibited tongues, arguing in favor of a pivotal role for the HH pathway in maintaining this epithelial cell population. This concept was further examined by immunostaining for the HH target Cyclin D1. We quantified Cyclin D1-positive cells in *apical* and *basal* papilla walls and in *perigemmal* cells, as we did for Ki67 expression (Fig 4H). In the *Gli2cKO* tongue, there was a trend to a reduced number of cells in the *apical* FP wall and in *perigemmal* cells. Cyclin D1-positive cells in *apical* and *perigemmal* regions were reduced by about 20% from Control (S3C, S3D and S3E Fig). Overall the data are similar to results with Ki67+ cell counts, but do not reach statistical significance.

Thus, GLI blockade leads to fewer proliferating cells in epithelial compartments that contribute to taste cells during homeostasis [27,32], suggesting that the loss of taste buds following HH pathway blockade is due at least in part to a deficiency in taste cell renewal.

### Apoptosis does not appear to contribute to loss of taste cells in GLI-inhibited mice

In an effort to gain insight into the mechanism of cell loss after HH blockade, we examined apoptosis in tongues from Control and GLI-inhibited mice. Immunostaining for cleaved caspase 3 did not reveal differences from Controls in any of our models (S3F and S3G Fig) and cell labeling was infrequent as predicted from other studies in taste bud [44,45]. TUNEL-positive cells are occasionally seen in epidermis and they are up-regulated after UV-irradiation [46] and also in tongue after X-ray irradiation [47]. We used H&E sections in Control and *Gli2cKO* tongues (S3H, S3I and S3J Fig) to directly compare with TUNEL reactions. There were multiple TUNEL-positive cells in Control and *Gli2cKO* FP epithelium (S3K, S3L and S3M Fig), with an accumulation of positive cells in more apical layers of *Gli2cKO* TYPE II and in TYPE III FP that had taste bud cell loss and acquisition of a conical, filiform-like cap (S3L and S3M Fig). Accumulated TUNEL-positive cells in the FP apex in GLI-inhibited models resembled those in filiform papillae in Control mice (S3H Fig FILI). These are unlikely to represent classical apoptotic cells because we did not detect cells expressing cleaved caspase 3; moreover, it is known that cell death in keratinocyte differentiation does not follow classic apoptotic pathways [48,49,50]. Overall, increased apoptotic cell death is not likely to be a principal factor in taste organ alterations during HH/GLI blockade.

### Innervation to fungiform papillae and taste buds is retained following epithelial GLI blockade

**Fig 5:**
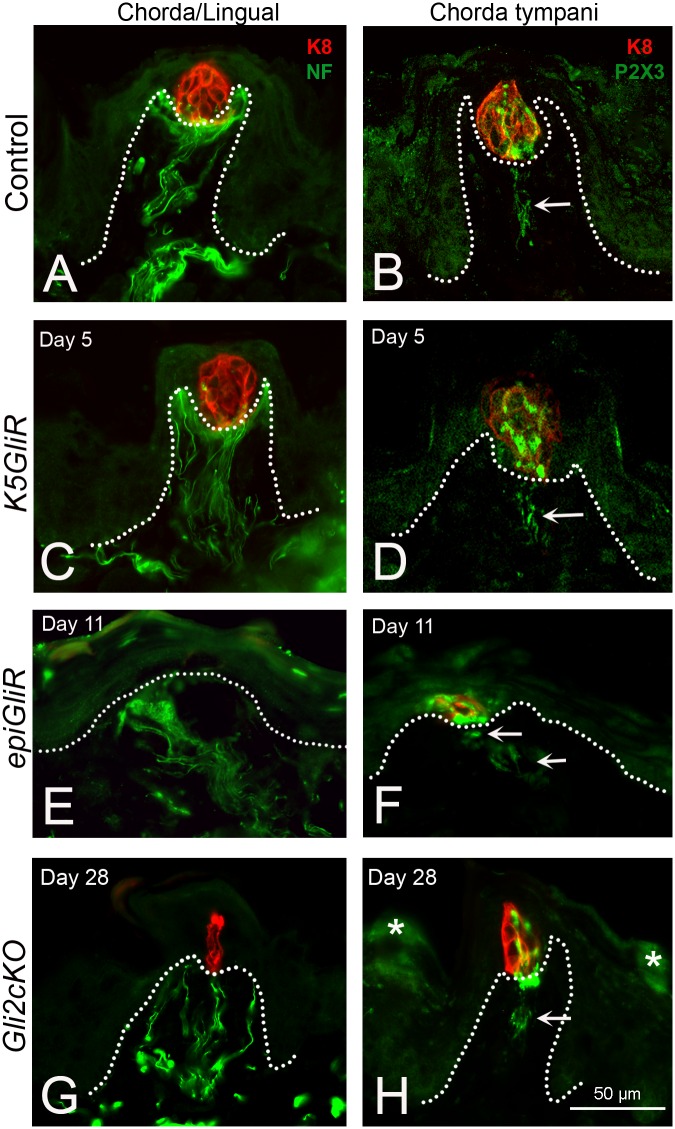
Innervation to fungiform papillae and taste buds is retained in epithelial HH/GLI suppression models. **A,C,E,G. Chorda/Lingual**: K8 immunostaining to label taste bud cells and neurofilament (NF) to label fibers of the chorda tympani/lingual nerve innervation to FP and TB. **A**. In Control the Chorda/lingual innervation is throughout the FP core and forms a dense ‘basket’ under the TB at the apex of the papilla core. With HH/GLI suppression at specified time points in *K5GliR* (**C**), *epiGliR* (**E**), and *Gli2cKO* (**G**) mice, NF-positive fibers are retained in the FP even though papillae are of atypical morphology and TBs are lost (E) or much reduced (G). At the illustrated time points in all models there are substantial morphological effects, as seen in Figs 1 and 2. **B,D,F,H**. **Chorda tympani**: K8 immunostaining to label taste bud cells and P2X3 to label fibers of the chorda tympani nerve innervation to FP and TB. **B**. In Control for Chorda tympani innervation the P2X3-positive label is within fibers in the papilla core (arrow) and within the TB. With HH/GLI suppression at specified time points in *K5GliR* (**D**), *epiGliR* (**F**), and *Gli2cKO* (**H**) mice, P2X3-positive fibers (arrows) are retained in the FP and TB cells even though papillae are of atypical morphology and TBs are lost (F) or much reduced (H). Scale bar in H applies to all panels. * in H indicate areas of nonspecific staining in the surface epithelial cells.

We demonstrated that FP and TB are disrupted or lost with HH/GLI blockade, and with fewer taste cells, SHH expression is much reduced in taste bud remnants. Further, proliferation was reduced in the TB perigemmal cells. Given the requirement for innervation in taste organ homeostasis [51], we examined innervation to the tongue and to taste buds specifically in *Gli2 repressor* (*K5GliR*, *epiGliR*) and *Gli2cKO* mice. Innervation to the tongue (combined chorda tympani/lingual nerve) and to taste buds specifically (chorda tympani only) was studied (Fig 5).

The connective tissue core of the FP typically contains neurofilament (NF)-positive fibers of the combined chorda tympani/lingual nerve that distribute to the taste bud and lateral wall regions of the papilla core (Fig 5A, Chorda/Lingual Control). A distinctive ‘basket’ of fibers encircles the basal lamina region just under taste bud cells. After 5 days of transgene activation in lingual epithelium of *K5GliR* or *epiGliR* mice, despite taste bud disruption (Fig 1C), there is a robust innervation within papillae as seen with NF label (Fig 5C, *K5GliR* Day 5). NF-positive nerve fibers are also detected in *epiGliR* mice despite severe disruption of taste buds and papillae (Fig 5E), and in *Gli2cKO* mice (Fig 5G).

FP innervation from the *chorda tympani* nerve to taste buds specifically, and *not* to the papilla walls, can be identified with immunostaining for P2X3 (Fig 5B, arrow, Chorda tympani, Control). P2X3 is strongly expressed in peripheral sensory, including gustatory neurons [52,53]. In contrast to NF, P2X3 immunostaining therefore detects chorda tympani nerve and also some fibers within the taste bud, with cells that co-express K8 (Fig 5B). Taste specific P2X3-positive innervation is retained in the papilla core of *K5GliR* mice and into the apical papilla epithelium and taste bud cell remnants (Fig 5D, *K5GliR*, Day 5, arrow). In day 11 *epiGliR* mice, P2X3-positive fibers are detected even though most K8-positive taste bud cells are lost (Fig 5F arrows). In FP of *Gli2cKO* tongues over a prolonged period of 28 days of doxycycline administration, taste nerves remain although FPs are disrupted and few retain intact TB remnants (Fig 5H arrow).

Importantly, we did not observe any re-patterning or displacement of lingual innervation from the FP to aberrant sites; that is, fibers remained directed to the appropriate ‘target’ region in *K5GliR*, *epiGliR* and *Gli2cKO* mouse tongues. In tongue, NF-positive fibers typically course within the reticular lamina propria under the epithelium (S4A Fig, Control arrows) and turn to innervate FPs (S4B Fig, Control). A similar pattern of nerves under the epithelium is retained in *K5GliR* and *epiGliR* tongues (S4C and S4E Fig arrows) and is in a directed course to densely innervate the FP (S4D and S4F Fig). Significantly, innervation is retained in FPs without K8-positive taste bud cells at a comparable extent to FPs with taste buds (S4B, S4D and S4F Fig).

The NF-positive bundles of the chorda/lingual nerve in the tongue body and coursing into the FP are surrounded by *lacZ*-positive, HH-responding cells (S4G, S4H and S4I Fig). Furthermore, S100-positive immunohistochemistry, to mark nerve Schwann cells, demonstrates a close association with HH-responding cells in the FP core (S4J Fig). Thus, any sources of SHH ligand from taste bud cell remnants or from other cell elements in the FP tissue core have direct access to nerve-associated HH-responding cells.

Our data demonstrate that overall there is no gross *elimination* or *misdirection* of innervation in tongue, FP and/or TB cells with HH pathway suppression over long periods and profound target organ disruption.

### Cells of the fungiform papilla core are retained and not grossly redistributed after HH/GLI repression

**Fig 6:**
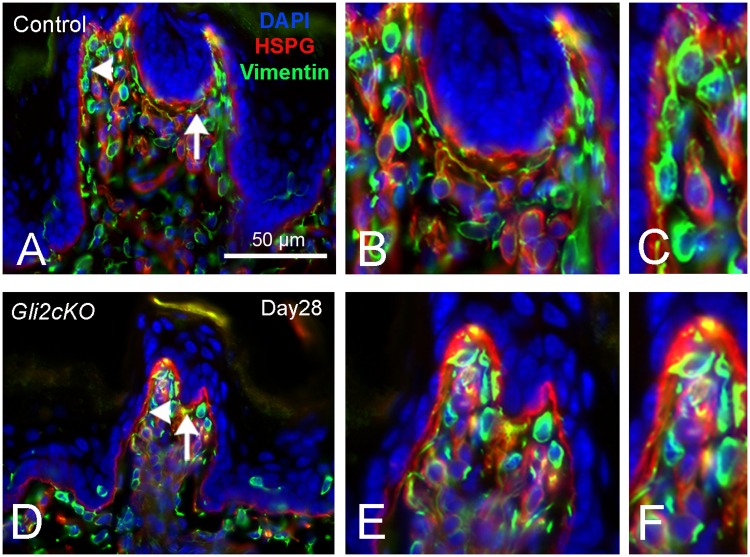
Stromal core of the fungiform papilla retains vimentin-positive fibroblasts with filopodia extensions after epithelial HH/GLI suppression. **A,B,C**. In Control FP, VM-positive cells (Vimentin) are throughout the FP core and in close association with basal lamina at FP walls and under the taste bud (A). B,C are enlarged images from A (regions with arrow and arrowhead). The epithelium is demarcated with DAPI. HSPG immunostaining delineates the basal lamina. Filopodia extend from vimentin-positive cells into basal lamina of FP walls (C). **D,E,F**. With HH/GLI suppression in *Gli2cKO* tongues, vimentin-positive cells remain in the FP core and filopodia extend into basal lamina of FP walls (D). E,F are enlarged images from D (regions with arrow and arrowhead).

Although taste buds and papillae are severely disrupted or lost in the *K5GliR*, *epiGliR* and *Gli2cKO* mouse tongues, FP-like structures can be identified that retain a somewhat rectangular shape, albeit more pointed apically, with epithelial down-growths (Figs 1 and 2). Also, HH-responding, *Gli1*^*lacZ*^-positive cells remain within the FP connective tissue (Fig 3). Because HH interacts with connective tissue cells and can be chemotactic [2,54], we used antibodies against vimentin, the principal intermediate filament of stromal fibroblast cells and a pan-fibroblast marker, to test for potential effects of HH suppression on FP stromal cells. In Control FP, vimentin-positive cells are: 1) in the central stromal core; 2) distributed along the papilla walls; 3) in the neurofilament-positive ‘basket’ niche just under the taste bud cells; and, 4) in cells that send filopodia extensions to contact the basal lamina (Fig 6A, 6B and 6C). In *Gli2cKO* tongues vimentin-positive stroma cells are not disrupted or redistributed but are retained in the basket region at the FP apex and have filopodia extending into the basal lamina (Fig 6D, 6E and 6F). Vimentin-positive cells were not observed within the taste bud in Control or *Gli2cKO* tongues; a few cells were observed in the epithelium. Overall there was no evidence for deregulated stromal cell activity in HH repression.

Other stromal cell types were labeled with antibodies for the macrophage marker, F4/80, and for smooth muscle actin (α-SMA). Labeled macrophages were in relatively small numbers in the FP core (S5A, S5B and S5C Fig) and there was no evidence of extensive macrophage infiltration associated with HH suppression. SMA-positive stromal cells were distributed in reticular lamina propria under the epithelium, within the filiform papilla core and the FP central core (S5D and S5E Fig) and were not numerous compared to vimentin-positive cells.

The data demonstrate that following epithelial repression of HH signaling and loss of taste organ integrity, major cell types in the papilla core are retained.

### The circumvallate papilla loses taste buds but retains size and shape and innervation after HH/GLI repression

**Fig 7:**
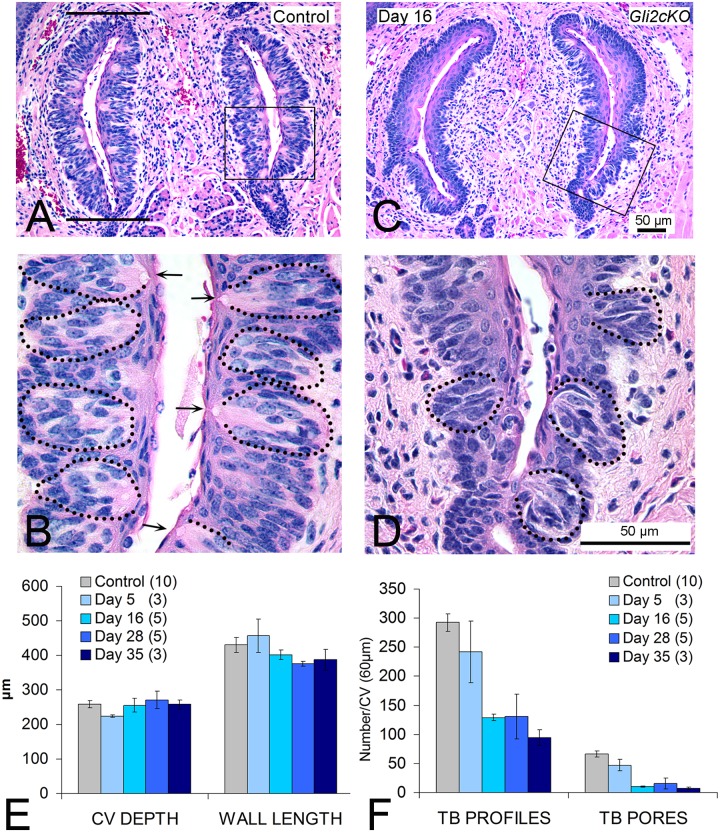
Circumvallate papilla structure is retained but taste buds are lost with conditional Gli2 deletion. Hematoxylin and eosin sections of the circumvallate papilla from Control (A,B) and conditional epithelial gene deletion, *Gli2cKO* (C,D) tongues. Papilla structure was measured from the number of serial sections that encompassed the papilla (depth) and length of each ‘wall’ marked by two bars in A. Whereas papilla shape is retained (A,C) numbers of taste buds are much reduced after gene deletion (B,D). Taste bud remnants (dotted ovals) or with complete pores (arrows) are numerous in Control papilla (B, enlarged from Box in A) but few are found with HH/GLI suppression (D, enlarged from Box in C). One scale bar applies for A and C; or B and D. **E,F**. Graphs for circumvallate structure (E) and numbers of taste bud profiles/remnants and pores (F) at increasing durations after *Gli2* gene deletion. CV depth and wall length did not change across time points in *Gli2cKO* tongues (E). Profile measures, used to indicate cell collections, were decreased by 28 days after *Gli2* gene deletion (F) and TB pore measures, to indicate complete TBs, also decreased (F). Taste buds with pores are essentially eliminated after 16 days gene deletion (F). Numbers of tongues studied at each time point after gene deletion are in parentheses in the legends. Full statistics for ANOVAs are presented in S1E and S1F Fig.

Whereas anterior tongue FPs have an ectodermal embryonic origin, the posterior tongue tissues and large circumvallate papilla (CV) have endodermal derivation [55,56] and could be affected differently by HH/GLI suppression. In contrast to the single TB in rodent FP, the CV contains a few hundred taste buds in dense physical juxtaposition in walls of the papilla trenches [57]. In Control and *Gli2cKO* mice, the general structure of the CV was similar (Fig 7A and 7C). To assess size of the CV we quantified length of CV walls (Fig 7A, bars on left wall) and computed depth of the CV from the number of dorsal to ventral serial sections. In *Gli2cKO* tongues these papilla measurements were not different from Control tongues across 5 to 35 days of gene deletion (Fig 7E). However, in contrast to the maintained overall CV structure, the numbers of TB profiles/remnants, or complete TBs with a taste pore, were markedly reduced compared to Control (Fig 7B, 7D and 7F). After 16 days of epithelial *Gli2* deletion, TB profiles were reduced by about 60% and full TBs (with pores) were almost eliminated (Fig 7F). (ANOVA data and posthoc analyses are presented in S1E and S1F Fig)

As in FP, NF-positive innervation to the CV, via the glossopharyngeal nerve, was not eliminated after epithelial gene deletion for 28 days which led to profound taste bud reduction (S6A and S6B Fig). Furthermore, comparable to results for FP, proliferating Ki67+ cells in the CV basal epithelium were substantially reduced after HH/GLI suppression (S6C and S6D Fig). To determine whether cell death was a major contributor to TB cell loss in the CV after HH inhibition, we performed TUNEL staining and did not discern a difference from Control tongue (S6E and S6F Fig). Again this is similar to the FP.

Similar to *Gli2cKO* mice (Fig 7F), in *K5GliR* tongues after 35 days of HH/GLI blockade, the number of CV taste pores was reduced to a mean of 10 compared to the control mean of 50 (S6G Fig). An even more profound effect on CV taste buds was observed in *epiGliR* mice: taste bud remnants and/or complete taste buds were essentially eliminated after 5 days (S7A, S7B, S7C and S7D Fig), and the CV shape was altered, with increased keratinization after 11 days (S7E and S7F Fig).

### HH responding cells and SHH expression are reduced in circumvallate papilla epithelium after K5-targeted GLI blockade

**Fig 8:**
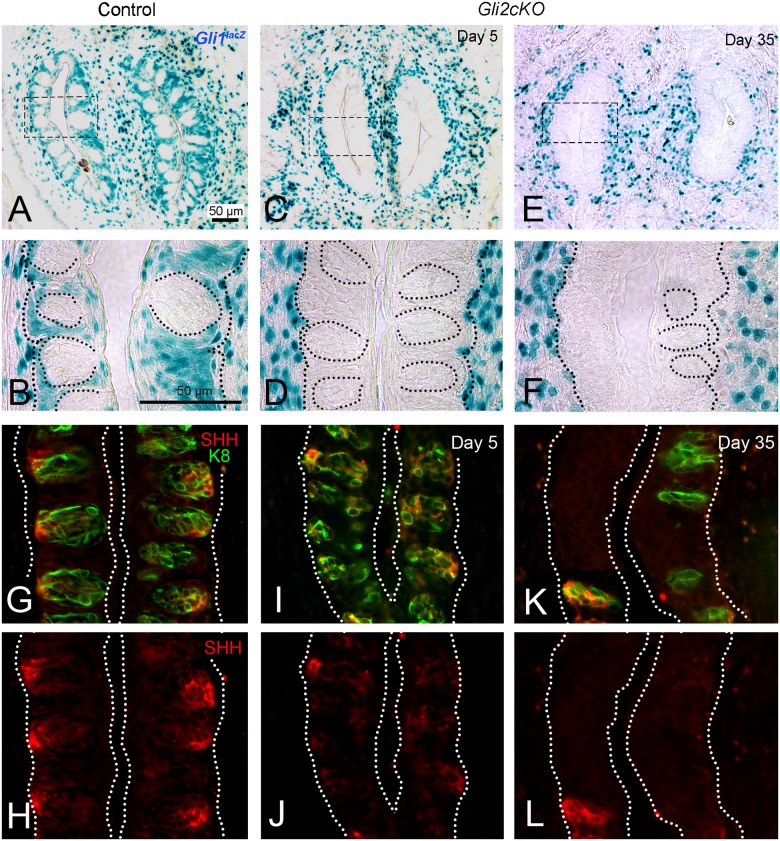
HH-responding cells are lost from circumvallate papilla epithelium but remain in stroma after epithelial Gli2 deletion, and taste bud size, and SHH expression, decrease with duration of HH/GLI repression. **A,B**. *Gli1lacZ*-positive cells in Control and **C,D;E,F** after gene deletion, in *Gli2cKO* CVs, at 5 and 35 days. B,D,F are enlargements for boxed regions in paired panels. **A,B**. In Control CV, HH- responding cells are in epithelial cells (perigemmal) surrounding the taste buds and in stroma of the papilla surround, including just under the basal lamina. **C,D; E,F**. From 5 days of gene deletion and continuing through 35 days, *Gli1lacZ*-positive cells are lost from the papilla epithelium but remain in stroma just under the taste bud- bearing epithelium. Dotted ovals are used to denote taste buds (B) or taste bud profiles/remnants (D,F). **G,I,K**. SHH and K8 Immunoreactions in Control, and at Day 5 and Day 35 after gene deletion illustrate reduction in K8-positive taste bud cells and associated loss of SHH expression. **H,J,L**. Same sections as those in merged panels (G,I,K) to illustrate SHH ligand alone in taste bud cells and remnants. After 35 days SHH expression is retained in reduced cell collections.

In Control mice carrying a *Gli1*^*lacZ*^ allele, X-Gal-positive HH-responding cells were detected in basal epithelial and perigemmal cells in the CV and in the stroma around taste bud-bearing CV walls (Fig 8A and 8B Control). However, in *Gli2cKO* mice, there was a sustained loss of HH-responding X-Gal-positive epithelial cells in the CV, as early as 5 days after gene deletion, with variable numbers of X-Gal-positive cells remaining in the surrounding stroma particularly near taste bud remnants (Fig 8C and 8D Day 5; Fig 8E and 8F Day 35).

In Control CV, SHH expression is clustered in the basal cell region of taste buds (Fig 8G and 8H). Associated with the loss of taste bud cells seen as early as Day 5 after gene deletion, there was a reduction in SHH expression within the taste buds of the CV (Fig 8I and 8J Day 5). With taste bud profiles or remnants some SHH-expressing cells remained, shown at Day 35 (Fig 8K and 8L Day 35).

In summary, the CV papilla epithelium was severely disrupted after HH/GLI blockade and was unable to sustain a full complement of TBs with pores, although papilla innervation remained. In association with taste bud cell loss, Ki67-positive basal epithelial cells were decreased, and epithelial HH-responding cells and SHH expression were reduced. These data indicate that both ectoderm-derived FP and endoderm-derived CV papillae require epithelial HH signaling for proper tissue maintenance.

### Loss of taste papillae and taste buds after HH/GLI blockade is reversible

**Fig 9:**
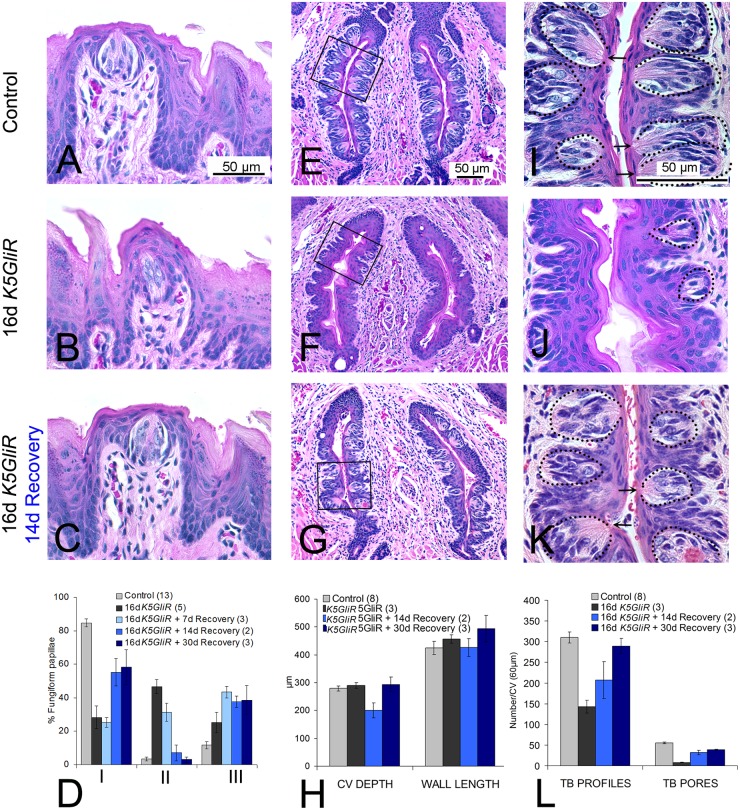
Recovery from effects of epithelial HH/GLI repression in fungiform and circumvallate papillae and taste buds. **A,B,C**. H&E sections of FP in Control (A), *K5GliR* after 16 days HH repression (B), and with 16 days HH repression followed by 14 days doxycycline withdrawal (C). **D**. Graph for FP recovery data. Whereas TYPE I Typical FP/TB are substantially reduced after 16 days of transgene activation, there is recovery after 14 days to more than 60% of TYPE I FPs. **E,F,G**. H&E sections of CV to illustrate papilla size and shape in Control (E) and in *K5GliR* tongue after 16 days HH repression (F) and with 16 days HH/GLI repression followed by 14 days doxycycline withdrawal (G). **H**. Graph for CV structure. There were no significant differences in CV size across Control, after HH/GLI repression, or recovery from HH/GLI repression. **I,J,K**. Enlargements, to illustrate taste bud pores and taste bud remnants, of boxed regions from Control (E), *K5GliR* after 16 days HH repression (F) and with 16 days HH repression followed by 14 days doxycycline withdrawal (K). **L**. Graph to demonstrate CV taste bud recovery. Taste bud profiles/remnants and taste buds with pores were much reduced after 16 days of HH repression but recovered to more than 60% Control levels with doxycycline withdrawal for 14 days, or up to 95% after 30 days. Dotted ovals are used to denote taste buds or taste bud remnants/profiles (I,J,K); arrows point to taste pores (I,K). Numbers of tongues at each time point are indicated in parentheses in graph legends.

To determine whether effects of epithelial HH/GLI blockade on papillae and taste buds were reversible, we induced transgene expression in *K5GliR* mice by treating with doxycycline for 16 days, and then withdrew doxycycline for a subsequent 7, 14 or 30 days. At 16 days of transgene expression, *K5GliR* FP were disrupted and taste bud cells were reduced or lost (Fig 9A and 9B). TYPE I FP/TB were reduced to about 25% of Control values and TYPE III Atypical FP/No TB papillae were about double those in Control, or 40% of all FP (Fig 9D). When doxycycline treatment was stopped to shutdown transgene expression, 7 days were not sufficient to induce recovery of FP/TB organs (Fig 9D). However, after treatment withdrawal for 14 days, epithelial integrity was restored (Fig 9C) and TYPE I Typical FP/TBs were recovered to about 80% of Control (Fig 9D). TYPE III Atypical FP/No TB papillae, on the other hand, also remained at a substantial proportion or about 40% of FP over 7–30 day recovery periods (Fig 9D), without apparent recovery. (All ANOVA data are provided in S8A Fig).

After 7–14 days recovery from 16 days of HH repression, X-Gal-positive, HH responding cells were again detected in perigemmal and basal epithelial cells in regenerated TYPE I Typical FP/TB (S9A, S9B and S9C Fig) but were only in the connective tissue stroma of TYPE III Atypical FP/No TB taste organs (S9G, S9H and S9I Fig). In 32% of TYPE II Atypical FP/Atypical TB after 7 days recovery there were some X-Gal-positive cells already appearing in the papilla epithelium (S9F Fig). Note that after 7 days recovery from HH repression, 25% of TYPE I Typical FP/TB have a distribution of *lacZ*-positive cells in FP epithelial walls and the stromal core (S9C Fig), whereas Type III Atypical FP/No TB have no *lacZ*-positive cells in the epithelium (S9I Fig).

To probe for recovery of proliferating cells we examined Ki67 expression in *apical*, *basal* and *perigemmal* cell regions of the FP. In tongues from Controls and mice with *K5GliR* transgene expression for 16 days, the results replicated those shown in Fig 4H for cell proliferation in *Gli2cKO* mice. In tongues from 16 day-induced *K5GliR* mice followed by 30 days treatment withdrawal, there was recovery of Ki67+ cell numbers in *apical* and *perigemmal* regions (S8G, S8H, S8I and S8J Fig). The extent of this recovery, significant in *perigemmal* cells, directly matched that for recovery of Type I FP/TB after treatment withdrawal (S8K Fig).

Similarly, CV papillae and taste buds also recovered after release of HH/GLI blockade. Sixteen days after GLI repression in *K5GliR* mice, the CV retained structural integrity compared to Control mice (Fig 9E, 9F and 9H). However, there was a clear and substantial loss of CV taste buds (Fig 9I, 9J and 9L), comparable to that seen in *Gli2cKO* mice (Fig 7). The CV taste bud profiles in *K5GliR* tongues were reduced to about 50% and pores were reduced to about 20% of Control values (Fig 9L). After 14 days off doxycycline treatment, the CV structure was still intact (Fig 9G and 9H) and taste bud profiles recovered to about 66% of Control values and to about 95% after 30 days (Fig 9K and 9L). Taste pores recovered to more than 80% of Control values after 14 or 30 days (Fig 9L). Compared to loss of HH-responding cells in the epithelium around the taste buds after HH repression (Fig 8), after 14 days recovery from treatment, X-Gal-positive cells were in the CV stroma and surrounding the taste buds (S10 Fig). (All ANOVA data are in S8B and S8C Fig)

With a shorter HH/GLI suppression period of 5 days, there was full recovery of TYPE I Typical FP/TBs, and of TB profiles and pores in the CV, within 14 days. Therefore with a shorter period of suppression and less extensive taste organ effects, recovery was complete (S8D, S8E and S8F Fig).

Thus, even after a prolonged period of HH/GLI repression, there was recovery of taste organ integrity in over half of FP papillae, and FP and CV taste buds, after only two weeks of doxycycline withdrawal. Therefore, during the period of epithelial HH/GLI blockade, progenitor cells that could reconstitute the papilla epithelium and taste buds upon reversal from HH/GLI repression survived or were rapidly re-formed. In TYPE III Atypical FP/No TB recovery was not detected, suggesting that with elimination of TB cells and therefore epithelium-derived HH ligand, progenitors for intact papillae and taste buds were irreversibly eliminated. The data suggest that HH-expressing taste bud cells are necessary for epithelial recovery from HH/GLI blockade.

## Discussion

Our findings using genetic blockade of GLI transcription factors identify a strict requirement for HH- responsiveness in epithelial cell populations of taste papillae during taste organ homeostasis, and uncover the likely mechanism underlying taste loss in cancer patients treated with systemic HH pathway inhibitors [29,30]. The rapid disappearance of taste bud cells and altered taste papilla integrity, followed by robust recovery when HH/GLI repression is stopped, underscores the plasticity of these tissues which are essential for nutrient identification and selection.

Typically, intense expression of the secreted ligand SHH is restricted within the taste buds, and via paracrine signaling can potentially affect HH-responding cells in epithelium and stroma of the taste organs [27]. Epithelial HH-responding cells include TB perigemmal cells and basal cells of the FP walls; stromal HH-responding cells are in the FP central core and are especially dense in the apical-most core. Stromal HH-responding cells co-localize with S100-positive, Schwann cells in lingual nerves and with vimentin-positive fibroblasts. Therefore, repressing HH signaling with *K5GliR*, *epiGliR* or *Gli2cKO* in K5-expressing cells of the mouse tongue, by disrupting SHH expression, could predictably affect activity in HH-responding cells in the papilla epithelium, in the perigemmal cells that surround the taste bud, in cells of the papilla connective tissue core, and innervating fibers.

In each of our four models using K5-targeted GLI inhibition, HH-responding cells were eliminated from taste papilla epithelium but retained in papilla stromal cells. The strikingly similar phenotypes seen with epithelium-specific GLI blockade strongly argue that the pivotal cell populations dependent on HH signaling are epithelial. In *GliR* and *cKO* mice, there is a progressive reduction in cell proliferation in the FP epithelium during the period of taste bud loss. The reduction is pronounced in the perigemmal region, but observed also in epithelium near the base of the FP in *epiGliR*. The epithelium at the FP base is a known proliferation niche that includes *Gli1*-positive, HH-responding cells that are multipotent progeny for the FP epithelial, perigemmal and taste bud cell lineages [27].

The HH pathway contributes to cell proliferation in development and cancer and can interact directly with the cell cycle machinery via upregulation of Cyclin D1 [58], which regulates progression from G1 to S phase. Whereas Cyclin D1 is considered a direct target of HH signaling, there is incomplete understanding of the cell biology that underlies the role of HH signaling in proliferation. Immunostaining for Cyclin D1 in Control and *Gli2cKO* mouse tongues yielded results in keeping with those for Ki67. Although these data are consistent with the concept that HH signaling regulates cell proliferation in taste organs through direct effects on the cell cycle, we cannot exclude the contribution of additional proliferation signals through secondary factors.

Our data suggest that epithelial and stromal cells have positional information based in cell-specific domains or niches in the FP that respond to a SHH organizing center, as in neural tube patterning [59]. In addition, in gustatory papillae the basal lamina microenvironment is contiguous with SHH-positive cells of the taste bud, with HH-responding cells of the perigemmal TB and the FP walls, with nerves, and with HH-responding, vimentin-positive stromal cells. Thus, there is direct opportunity for epithelial/stromal cell interactions to regulate FP and TB maintenance via HH signaling control in basal lamina domains.

### Recovery from HH signaling suppression

Our data demonstrate effects of HH signaling block in K5-expressing cells in two very different gustatory papillae: the FP with a single taste bud in the apex and the CV with a few hundred taste buds that are dense and in physical juxtaposition in walls of the papilla trenches [57]. On withdrawal of doxycycline to stop transgene expression in *K5GliR* mice, a large proportion of FP and CV epithelia and taste buds recover. In FP perigemmal cells, the Ki67 cell expression that is reduced after HH/GLI suppression also recovers. Therefore TB progeny are not eliminated in HH signaling repression in the epithelium of these papillae but are poised to regenerate TBs, with innervation and papilla organ connective tissue core elements already in place, once HH/GLI signaling is restored. SHH-positive cells within TBs are proposed as a possible obligate stage in differentiation of all TB cell types, as post-mitotic precursors not stem cells [35].

However, we find that a subset or about 40% of TYPE III Atypical FP/No TB taste organs do not recover after HH/GLI blockade. Because these FPs lack TBs, they lack SHH ligand in the epithelium and do not attain reconstitution of HH-responding cells in the papilla epithelium. Therefore we suggest that epithelium-derived HH is essential to maintain and re-activate TB cell progenitors which may reside within TBs.

### Pharmacologic blockade of HH signaling and epithelial HH suppression

We note that after 14–30 days to restore HH/GLI signaling after HH repression, the recovery of Typical FP/TB and CV taste buds is substantial but incomplete in the FP/TB. Notably, in a pharmacological block of systemic HH signaling with the HH pathway inhibitor drug LDE225, there also is substantial loss of Typical FP and taste buds after 16 days [31]. These experiments uncovered the likely mechanisms underlying taste loss in cancer patients treated with systemic HH pathway inhibition [29,30]. Although with LDE225 pathway inhibition the taste buds and associated SHH were lost, a robust innervation remained within aberrant fungiform papillae. As in our genetic models to block epithelial HH signaling, there also is recovery after 14 days without the inhibitor drug, of about 50% of Typical FP/TB [60]; and these typical organs demonstrate a full complement of X-Gal-positive, HH responding cells in papilla epithelial walls and perigemmal cells. However, in LDE225 HH inhibition, and all genetic models, about 40% of taste organs are Atypical FP/No TB, and in these papillae HH-responding cells are only in the papilla core. We are studying the nature of the FP/TB that apparently are incapable of regeneration.

### Papilla and taste bud disruption in epithelial HH repression does not eliminate nerves or alter innervation pattern

Whereas we do not rule out a possible decrease in nerve fibers, at the light microscopic level there is no *general elimination* or *misdirection* of innervation in anterior tongue, FP and/or TB cells even after epithelial HH pathway suppression over long periods. Also, innervation remains robust in the CV in *Gli2* repressor and *cKO* mice. Because SHH is much reduced in the taste bud, in parallel with the loss of SHH-expressing taste bud cells, the retained innervation indicates that SHH is not a major, taste bud, target-derived maintenance factor for innervation to the papilla and TB.

Further, because P2X3 fibers remain in FP in *Gli2* mutant and deletion tongues there is apparently not a major destruction of geniculate ganglion neurons that project via the chorda tympani to taste buds. In a pharmacologic block of HH signaling with the HH pathway inhibitor drug LDE225 we also demonstrated that although taste buds were lost, thus reducing available taste bud-derived SHH, a robust innervation remained to taste bud cell remnants and aberrant fungiform papillae [31].

However, we have localized HH-responding, *Gli1*^*lacZ*^-positive cells adjacent to nerve bundles and S100-labeled Schwann cells within the FP. Furthermore, within the body of the tongue *Gli1*^*lacZ*^-positive cells are seen along the perimeter of large bundles of the chorda tympani/lingual nerve. Overall our data put *Gli1*^*lacZ*^-positive cells in contiguity with nerve fibers and nerve-associated Schwann cells, possibly even in cells of the perineurium [61]. We propose that in addition to SHH in taste bud cells, there are possible sources of SHH from the geniculate ganglion and trigeminal ganglion, and via innervating nerve fibers could potentially provide local SHH ligand to *Gli1*^*lacZ*^-positive HH-responding cells in the taste papilla organ. SHH has been reported in trigeminal ganglion neurons in studies of pulp innervation [62]. If sensory nerve endings are a source of HH ligand from trigeminal or geniculate ganglia these could activate HH signaling in stromal cells of the FP core, as seen in hair follicle innervation [3]. There is, then, ligand from SHH released from taste buds in epithelium, much reduced with HH suppression; and, potentially from trigeminal and geniculate ganglia at nerve endings within the FP. Thus, HH-responding cells in multiple domains of the papilla organ could have access to ligand from at least two sources.

Not only do we find that SHH in taste bud cells is apparently not a major target-derived support factor for FP/TB lingual innervation but also, even the sustained lingual and FP innervation is not sufficient to maintain or regenerate intact taste buds in the face of epithelial HH-repression. Innervation is necessary to maintain taste buds but on anterior tongue of mouse with combined chorda tympani and lingual nerve cut a number of taste buds remain [51]. Therefore whereas nerves are essential to transmit taste responses to the CNS and to maintain taste buds, there apparently is not a *complete* or *sole* sensory, chorda tympani nerve dependence of all adult FP taste bud cells. In mice with SHH over-expression, K8-positive, taste bud-like cells can form in lingual epithelium outside of FPs but these are not innervated and therefore are not able to transmit taste sensation [28]; this indicates a taste bud cell-like phenotype in the absence of taste innervation and chemosensory function. Therefore, the sensory nerve dependence of taste bud cells is complex [63].

### Stromal cells are retained in HH suppression

The importance of stromal cells in the papilla core, adjacent to taste bud cells and their supporting epithelium, has been essentially ignored in the taste field. Stromal fibroblasts with the intermediate filament vimentin are important in cell adhesion, migration and signaling [64] and SHH can be chemotactic and interact with fibroblasts in SHH trafficking [54]. There also is ample evidence that HH signaling regulates fibroblast activity, e.g., in kidney interstitium [65], in lung [2,5] and during pancreas tumorigenesis [6]. However, despite the profound alterations in taste organs that arise after blockade of HH/GLI signaling in epithelial cells, immunostaining for fibroblasts did not reveal appreciable differences compared to Control mice. This is in contrast to mesenchymal cell proliferation when HH signaling is downregulated after injury in adult lung epithelial cells [2]. Nor did we observe a massive influx of macrophages in the papilla core. The retention of multiple cell populations, and nerve endings, in the papilla core, despite disruption of overall papilla structure, may provide a crucial microenvironment needed for recovery of taste organs after HH/GLI blockade.

### Taste homeostasis and HH signaling

Sensory homeostasis demands balanced cell physiology, and physiological adjustments in major signaling pathways can alter taste function and create risk for diet selection, toxin avoidance and proper nutrition. We showed that HH/GLI signaling is essential to preserve homeostasis in taste papillae and resident TBs; this pathway operating in peripheral taste organs is, therefore, crucial for taste sensation and nutrient regulation. Our findings contribute to understanding the biological basis of taste alterations in patients who take HH Pathway Inhibitors (HPIs) for treatment of basal cell carcinoma [29,30]. The responses to genetic HH/GLI repression that we observed in FP have a similar time course to FP/TB alterations in mouse tongue after HPI administration [31]. HH repression in the lingual epithelium leads to profound loss of taste buds but nerves and connective tissue cells are maintained within taste papilla organs. Therefore, taste bud progenitor cells are strictly dependent on epithelial HH signaling, and can function to regenerate taste buds when HH signaling is restored as long as some residual TB cells remain. However, if HH and HH-responding cells are eliminated from the fungiform papilla epithelium through taste bud loss, papillae and taste buds do not recover from HH suppression. This suggests that HH in taste bud cells, acting through paracrine signaling to responding cells, is in a principal role for taste bud and papilla maintenance and restoration. To keep taste bud cell homeostasis at a necessary steady state for sensory function, epithelial HH/GLI signaling is required.

## Methods

### Animals

Maintenance of mice and all experimental procedures were conducted in accordance with NIH guidelines and were approved by the University of Michigan Institutional Animal Care and Use Committee (protocols: PRO00006464, BLA; PRO00006657, AAD; PRO00005851, CMM).

### Mouse strains and transgene induction

*Gli1*^*lacZ/+*^: mice were used to indicate cells responding to HH/expressing the target gene *Gli1*.

Mice were maintained on mixed backgrounds.

*K5-rtTA;tetO-Gli2ΔC4;Gli1*^*lacZ/+*^*(K5GliR)*: doxycycline-inducible, reversible, dominant-negative inhibition of Hh/Gli target genes in K5 expressing, basal epithelial cells (littermates negative for *K5rtTA* and/or *tetO- Gli2ΔC4* were used as controls).

*K5-Cre;R26-LSL-rtTA;tetO-Gli2ΔC4;Gli1*^*lacZ/+*^*(epiGliR)*: doxycycline-inducible, dominant-negative inhibition of Hh/Gli target genes in K5 expressing cells and their progeny (littermates negative for *K5rtTA* and/or *tetO- Gli2ΔC4* and/or *R26-LSL-rtTA* were used as controls).

*K5-rtTA;tetO-Cre;Gli2*^*fl/fl*^*;Gli1*^*lacZ/lacZ*^ and *K5-rtTA;tetO-Cre;Gli2*^*fl/fl*^;*Gli1*^*lacZ/+*^*(Gli2cKO)*: doxycycline-dependent, Cre-driven deletion of *Gli2* in K5-expressing cells, heterozygous or null for *Gli1* (littermates negative for *K5rtTA* and/or *tetO-Cre* were used as controls).

*K5-rtTA;tetO-Cre;Gli2*^*fl/fl*^*;Gli1*^*lacZ/lacZ*^ *(Gli2cKO;Gli1KO)*: a Gli2/Gli1 double deletion in K5-expressing cells (littermates negative for *K5rtTA* and/or *tetO-Cre* with *Gli1*^*+/+*^ were used as controls).

*K5-rtTA* mice [66] were obtained from Adam Glick (Pennsylvania State University). Each of the following strains was obtained from Jackson Labs: *Gli1*^*lacZ/lacZ*^ (Stock No: 008211) [18]; *Gli2*^*fl/fl*^ (Stock No: 007926) [67]; *tetO-Cre* (Stock No: 006224) [68]. *tetO-Gli2ΔC4* mice were generated using a mouse *Gli2ΔC4* cDNA [4,40] inserted into pTet-Splice using standard molecular cloning techniques (Ermilov et al in preparation).

Doxycycline was administered in chow at 1 g or 6g doxycycline/kg chow (Bio-Serv), for specified periods. Once started, doxycycline treatment for conditional, epithelium-specific gene deletion studies was continuous throughout the treatment period. For recovery studies using *K5GliR* mice, doxycycline administration was stopped to extinguish *Gli2* expression. Animals were maintained on normal chow to monitor recovery from HH/GLI blockade.

For each experiment and time point, observations were made in at least 3 experimental and 3 littermate control mice, with noted exceptions for very long experimental periods. Actual animal numbers are included in all graphs.

### Tissue dissection and processing

#### Tissue preparation

Tongues on mandibles were removed from mice euthanized with CO2, fixed for 2 hours at 4°C in 4% PFA in 0.1M PBS, and then tongues were dissected from the mandible. Tongues were cut anterior to the intermolar eminence, and the anterior portion was bisected into two halves for sectioning each in the sagittal plane. The posterior tongue was cut just anterior to the foliate papillae and at the posterior oral tongue border, to include the full circumvallate papilla for sectioning in the horizontal plane parallel to the surface of the tongue. The three tongue pieces were returned to fixative for another 1 to 3 hours and then one half of the anterior tongue was cryoprotected overnight with 30% sucrose in PBS and frozen in O.C.T. Serial sagittal sections were cut at 10 μm and mounted for immunostaining [24,25]. The other anterior half tongue and posterior tongue piece remained in fixative overnight for subsequent paraffin serial sectioning at 6 μm, sagittal plane (anterior half) or horizontal plane (posterior piece with CV), and stained with H&E. For X-Gal staining tongue halves were fixed for a total time of 2 hours.

#### Immunostaining and imaging

Immunoreactions were according to routine procedures [27,31]. Primary antibodies were: Goat anti-Shh (AF464, 0.1 μg/ml, R&D Systems); Rat anti-Keratin 8 (TROMA-1, 1:1000, Developmental Studies Hybridoma Bank); Rabbit anti-Neurofilament-H (NB300-135, 1:1000, Novus Biologicals); Rabbit anti-P2X3 (NB100-1654, 1:2000, Novus Biologicals); Mouse anti-p63 (SC8431, 1:100, Santa Cruz); Rabbit anti-Ki67 (Ab16667, 1:250, Abcam); Rabbit anti-Cyclin D1 (Rb212P, 1:200, Thermo Scientific); Rabbit anti- Cleaved Caspase 3 (9661, 1:250, Cell Signaling); Rabbit anti- S100 (Ab868, 1:1000, Abcam); Chicken anti-Vimentin (AB5733, 1:1000, Millipore), Rabbit anti-Vimentin (5741, 1:1000, Cell Signaling); Goat anti- E-Cadherin (AF748, 1:1000, R&D Systems); Rat Anti-F4/80 (AB6640, 1:500, Abcam); Mouse anti-human Smooth Muscle Actin (M0851, 1:100, Dako); Rat anti-HSPG (Perlecan) (NB600-583, 1:5000, Novus Biologicals). For Shh, Ki67, p63 and P2X3, antigen retrieval was used by immersing slides in boiling citrate buffer, pH 6, for 3 Min. Secondary antibodies were Alexa Fluor conjugates 488 or 568 (Life Science Technologies, 1:500). Sections were counterstained with DAPI solution. As negative controls, sections were processed with omission of primary antibody, or with comparisons to known positive tissues. Images were acquired with a Nikon Eclipse 80i microscope and figures assembled with Adobe Photoshop.

For X-Gal staining, frozen sections from experimental and control *Gli1*^*lacZ/+*^ reporter mice were dried, rehydrated and transferred into X-Gal solution [27]. For EdU (Life Technologies) cell labeling, mice were injected i.p. at 50mg/kg EdU dissolved in 0.9% sodium chloride, 3 hours before sacrifice. EdU labeling was detected with the Click-iT kit (C10339, Invitrogen) using manufacturer’s protocol. TUNEL staining was done with the Millipore Kit (S7165).

### Quantification

#### Taste papillae and taste buds

*Fungiform Papilla (FP) and Taste Bud (TB) Types*. On each half tongue in H&E serial, sagittal sections, 600 μm of the middle tissue sections were analyzed, to exclude sections with FP in varied orientation on the lateral border or on the generally papilla-free tongue midline. Fungiform papillae (FP) and Taste Buds (TB) were categorized in three Types in hematoxylin and eosin sections, for quantifying effects. TYPE I: Typical Fungiform Papilla and Typical Taste Bud: Papillae are rectangular, with a multi-layered epithelium that is thin at the apex and includes a single taste bud. Cells of the taste bud are oriented and cluster apically around a taste pore. A broad connective tissue core includes stromal cells, nerves, vessels and matrix. TYPE II: Atypical Fungiform Papilla and Atypical Taste Bud: The papilla is somewhat misshapen and there is an increase in most superficial cornified cell layers. The taste bud is narrow or with reduced cells, usually lacks a taste pore, and is identified as a collection of cells that are not oriented. TYPE III: Atypical Fungiform Papilla and No Taste Bud. The papilla does not maintain a well-defined ‘rectangular’ core, has a pointed or conical apical ‘cap’ of cornified cells, and has either no or extremely few taste bud cells. These categories are illustrated in Fig 1B. Data are reported as percentage of FP/TB Type I, II or II, relative to full FP/TB counts (about 30 FPs per tongue).

*Circumvallate papilla (CV)*. The single posterior CV in rodent is a dome-shaped structure surrounded by a ‘moat’ and an epithelial wall and contains a few hundred taste buds in rodent that are dense and in physical juxtaposition in papilla walls [57]. The CV was serially sectioned (6 μm) at a plane horizontal to the surface of the tongue to encompass the entire papilla with all taste buds in complete section. We measured CV depth and wall length, and counted taste bud profiles or remnants, and complete taste buds from analysis of serial sections, as follows. After determining the full number of sections occupied by the papilla, the middle 10 sections were selected and all taste bud profiles or remnants of cell collections in every section were counted, in all four CV wall sections (Fig 7). Within these 10 middle sections each taste bud with a complete pore also was counted. Measurement of CV ‘depth’ was calculated as the number of all sections with any indication of CV walls times 6 μm. CV wall length was determined in the middle CV section by measuring the length of each of the four walls, as shown in Fig 7A, and calculating the average.

*Proliferation*. We assessed proliferation with antibodies to Ki67, p63 and Cyclin D1; and for S-phase labeling with EdU. To quantify proliferation in FP we used Ki67/E-Cadherin or cyclin D1/E-cadherin double immunoreactions that identified proliferating cells and clearly delimited the epithelium. For Ki67 analysis, we studied one experimental and one control tongue each for the *epiGliR* model after 5 days induction, and for the *Gli2cKO* model after 16 days. These were time points with loss of 90% or more Typical FP/TB structures. For each tongue we selected 8 FP that were on the anterior 1/3 of the tongue, were in complete serial sections, and were not mis-oriented. Images were captured for sections through the identified center of each FP. Papilla walls were divided into apical and basal halves after measuring the full length of the basal cell compartment from apical to basal extent, for the left and right walls (seen in Fig 4F). Ki67 positive cells were counted in each wall, *apical* and *basal* halves; then counts for left and right walls were added for each FP. We separately counted Ki67+ cells in the *perigemmal* region. For Cyclin D1 analysis, we studied two Control and two *Gli2cKO* tongues at 24 days after gene deletion. We used the protocol for Ki67 analysis and sampled 9–11 FP in two tongues for counting. To evaluate proliferation in the CV we used Ki67/K8 double immunoreactions and assessed the distribution pattern for Ki67+ cells in three Control tongues and three tongues from *Gli2cKO* mice at 16 days after gene deletion.

### Cell death

The terminal deoxynucleotidyl transferase dUTP nick ending labeling assay (TUNEL; Millipore Kit S7165) was used with manufacturer protocol to detect apoptotic cell nuclei. We studied TUNEL-positive cells in FP in one experimental and one control tongue from *Gli2cKO* mice at 28 days after deletion; and for CV in two experimental and two control tongues from *Gli2cKO* mice at 16 days after deletion and assessed distribution patterns in papilla and surrounding epithelium. We studied Cleaved Caspase 3 in FP in one experimental and one control tongue each for the *K5GliR* model after 5 days and for the *epiGliR* model after 11 days induction. Cleaved Caspase 3 in the CV was studied in one Control tongue and one *Gli2cKO* at 16 days after deletion.

### Innervation

Papilla innervation was analyzed in immunoreactions with antibodies to Neurofilament Heavy (NF200) and for specific chemosensory innervation with an antibody to P2X3. We studied 1–2 experimental and one control tongue each for *K5GliR* and *epiGliR* models across 3 time points, one control and one experimental tongue from *Gli2cKO* mice at 28 days post induction. Innervation patterns were analyzed qualitatively to identify nerve fibers in the “basket” region just under the taste bud region (using NF) or within the taste bud (using P2X3) and fibers directed through the middle of the papilla core.

### Stromal cell activity in FP

To identify fibroblasts within the FP core and discern how these cells are associated with the epithelium or with basal lamina we used double immunoreactions for vimentin and heparan sulfate proteoglycan (HSPG) to delineate the basal lamina. We studied 8 FP each in *Gli2cKO* tongues, one experimental and one control, at 28 days post induction. For each of the FP we captured images of four serial sections (or 8 X 4 sections per tongue). We counted FP stromal cells that were in basal lamina contact, that is, contiguous to/touching or crossing/within the basal lamina in each tongue. In these same sections we counted vimentin-positive cells that were in the core of the papilla, that is, in the stroma not in basal lamina contact. We also counted all Vimentin-positive cells within the epithelium for each FP. To ensure that vimentin-positive cells were not macrophages or smooth muscle actin-positive cells we used immunoreactions for F4/80 or αSMA respectively.

### Statistics

All data in figures are presented as means and standard errors. For analysis of FP types and CV measures, across time periods, we used One Way Analysis of Variance (ANOVA) for each papilla type, with the Least Significant Difference posthoc test, and a significance level of p<0.05. Numbers of tongues/FPs are in graphs. In Supplemental Figs 1 and 9, complete F and p values are presented for FP and CV quantification data/graphs. For all ANOVA, we pooled data from Control mice for the comparison group against Experimental time points. For *Gli2cKO*;*Gli1KO* FP analysis, to overcome small sample sizes at some time points, we compared pooled Control (n = 6) with pooled data from doxy-treated Day 16,28 (n = 4) and from Day 35,45 tongues (n = 4) but all time points are shown in figures.

In analysis of cell proliferation and stromal cells numbers, the independent samples t test, with Levene’s test for equality of variance, was used to compare differences between treatments (significance level = p ≤ 0.05).

## Supporting Information

S1 Fig ANOVA data for effects in fungiform and circumvallate papillae and taste buds. **A,B,C,D**. ANOVA data with posthoc comparisons for FP effects in Figs 1 and 2 after HH/GLI repression (A, *K5GliR*; B, *epiGliR*) and gene deletion (C, *Gli2cKO*;D, *Gli2cKO;Gli1KO*). **E,F**. ANOVA data with posthoc comparisons for effects of *Gli2cKO* in Fig 7 on CV structure and numbers of taste bud profiles/remnants or taste pores (E,F). Significance levels are indicated (*p<0.05; **p<0.01; ***p<0.001).
(TIF)

S2 Fig HH-responding cells are lost from fungiform papilla epithelium in HH suppression models and taste bud cell collections are much reduced after long duration HH/GLI suppression in *K5GliR* mice. **A**. X-Gal staining to detect β-gal-positive, HH-responding cells in Control *Gli1*^*lacZ/+*^, and *K5GliR*, *Gli2cKO*, and *Gli2cKO;Gli1KO* mice. After transgene activation there is loss of detectable HH signaling in epithelium, with *lacZ*-positive cells in the FP stroma only. **B**. Compared to Control, very few taste bud remnants are observed after 35 days HH/GLI repression in *K5GliR* tongues and SHH ligand is reduced, associated with the taste cell loss. Scale bar applies to all images.
(TIF)

S3 Fig Distribution of proliferating cells and Caspase 3-positive and TUNEL-positive cells in fungiform papilla in tongues with HH suppression. **A,B**, FP immunostained for p63 and E-cadherin to label the epithelium, in Control (A) and *Gli2cKO* tongue (B). p63-positive cells are continuous and apparently homogeneous throughout the basal epithelial cells of the tongue and papillae in control tongue and after HH signaling repression. **C,D,E**. Cyclin D1 immunostaining in FP in Control (C) and *Gli2cKO* (D) tongues and graph (E) for cell counts of Cyclin D1-positve cells in APICAL, BASAL and PERIGEMMAL regions of the FP (compare to Fig 4H for Ki67-positive cells). Cyclin D1-positive cells tended to decrease in APICAL and PERIGEMMAL regions in FP from tongues after HH suppression compared to Control, but there were no significant differences. Numbers in parentheses indicate number of FP analyzed. **F,G**. Cleaved Caspase 3-positive cells (CC3, arrows) with Ecadherin (Ecad) immunostaining indicates infrequent CC3 label in Control and *K5GliR* FP. **H,I,J**. H& E sections to illustrate phenotype for Control (H), and two examples of disrupted FP and taste buds after gene deletion (*Gli2cKO*) for 28 days (I,J). The taste bud or taste bud remnant is circled in all images. **K,L,M**. TUNEL staining in Control (K) and two examples for *Gli2cKO* tongues to label dying cells (L,M), with Ki67 immunoreactions for proliferating cells. Taste buds or remnants are circled in each image. In Control FP (K) TUNEL-positive cells are in suprabasal regions of the epithelium and FP apex. In filiform papillae (FILI) there are accumulated TUNEL-positive cells in suprabasal apical regions. In two examples from *Gli2cKO* tongues of disrupted FP and taste bud cell remnants (L,M) compared to Control the extent of TUNEL stained cells at the extreme papilla apex, which becomes conical and heavily keratinized, has apparently increased somewhat. Dotted lines demarcate the border of the superficial epithelial cells that are beneath the keratinized surface squames. Scale bar in M applies to all images.
(TIF)

S4 Fig Innervation retained in directed pattern in tongues and papillae with HH/GLI suppression. **A,B; C,D; E,F**. Anterior tongue sections and sample FP from Control (A,B), *K5GliR* (C,D) and *epiGliR* (E,F) tongues after 5 days. K8 labels taste bud cells and neurofilament (NF) labels lingual innervation. With HH/GLI repression, nerves are not lost or redirected but track under the epithelium and turn to densely innervate FPs (A,C,E, arrows). Even FPs without taste bud cell remnants are innervated (D,F). **G,H**. *Gli1lacZ-positive cells* and NF immunostaining in Control tongue illustrate that HH-responding cells are in direct association with, or lining, nerves in the tongue body (G) and going into the FP (H). **I,J**. NF labeled fibers (I) and S100-positive Schwann cells (J) are in direct association with *Gli1 lacZ*-positive, HH-responding cells in FP.
(TIF)

S5 Fig Fibroblasts and other cells of the fungiform papilla stroma. F4/80 immunostaining for macrophages, with vimentin reactions, in Control (A) and conditional *Gli2* deletion (B) tongues, demonstrate absence of macrophage invasion in FP with HH suppression. As a positive control for the macrophage labeling we used tongues with lingual nerve cuts, a procedure known to increase macrophage invasion (C). **D,E**. To label myofibroblasts we used immunostaining for smooth muscle actin (αSMA) and identified cells within the stroma core of filiform (D) and fungiform (E) papillae. Ecad and DAPI demarcated the epithelium.
(TIF)

S6 Fig Circumvallate papilla innervation is retained, cell proliferation is decreased, and TUNEL staining is not appreciably altered in *Gli2cKO* tongues. **A,B**. Control circumvallate papilla (A), with K8 immunostaining to label taste bud cells, has extensive innervation around papilla walls as seen with NF immunostaining. (Asterisk in A indicates nonspecific fluorescence.) With conditional gene deletion after 28 days in *Gli2cKO;Gli1KO* tongue, nerves are retained around the circumvallate papilla walls (B). Taste bud cells are much reduced. **C,D**. Control papilla (C), with K8 to label taste bud cells, and Ki67 immunostaining to label proliferating cells illustrates Ki67-positive cells in basal epithelium of the papilla. In *Gli2cKO* tongue (D), the taste bud cells and Ki67-positive cells are much reduced. **E,F**. In Control (E) and *Gli2cKO* (F) papilla, TUNEL staining labels cells seen in suprabasal regions near the luminal surface of the papilla. The extent of TUNEL-positive staining is not noticeably different in Control and *Gli2cKO* CV. Dotted lines indicate the extent of the papilla epithelium. In panel D, an ‘L’ denotes the luminal side of the papilla epithelium that applies to C,D,E,F. **G**. Data Table for results comparing CV in Control tongues with *K5GliR* tongues after 35 days HH suppression. Whereas CV size is not different, taste pores are reduced in *K5GliR* CV (t = 14.7, p = 0.005). Scale bar in B applies to A and B; scale bar in F applies to C,D,E,F.
(TIF)

S7 Fig With HH/GLI repression throughout the epithelium all taste buds are eliminated from the circumvallate papilla. **A,B**. H&E sections through the mid-region of circumvallate papillae from two Control tongues, to illustrate complete papilla structure and epithelium replete with taste buds. **C,D**. Mid-region of circumvallate papillae from two tongues with HH/GLI repression, at 5 days after *epiGliR* transgene activation. **E,F**. Mid-region of circumvallate papillae from two tongues, at 11 days after *epiGliR* transgene activation. Taste buds are not observed within the papilla epithelium. Scale bar in F applies to all images.
(TIF)

S8 Fig Recovery from effects of HH/GLI repression in fungiform and circumvallate papillae. **A,B,C**. ANOVA data for effects of recovery at 7 to 30 days after HH/GLI repression for 16 days in FP (A) and CV (B,C), presented in Fig 9. **D,E,F**. Effects for 5 days HH/GLI repression and subsequent 14 days of recovery in FP (D) and CV (E,F). After a shorter repression duration of 5 days, compared to A,B,C, the recovery to TYPE I Typical FB/TB is essentially complete (D). CV depth and wall length are not different in any groups (E). Recovery to full numbers of TB profiles and pores is seen after 14 days (F). Numbers of tongues at each time point are indicated in parentheses in graph legends. **G,H,I,J,K**. Ki67-positive, proliferating cells in Control FP (G), *K5GliR* FP after 16 days HH suppression (H) and *K5GliR* FP after 30 days recovery from 16 days of HH suppression (I). Cell counts in APICAL, BASAL and PERIGEMMAL regions of the FP are graphed (J). Numbers of FP at each time point are indicated in parentheses in graph legend. The extent of recovery of Ki67-positive cells is directly comparable to that of Type I Typical FP/TB (K; data are extracted from Fig 9D, and S8A Fig).
(TIF)

S9 Fig Fungiform papilla recovery of epithelial HH-responding, *Gli1lacZ*+ cells in Typical and Atypical FP/TB, not in Atypical FP/ No TB, after HH/GLI repression. The distribution of HH- responding cells, *Gli1*^*lacZ*^ positive, after Recovery matches presence of Typical or Atypical taste buds in specific fungiform papillae. **A,B,C**. Examples with percentages of representation for TYPE I, Typical FP/TB, in tongues of Control mice (A, 84%), after 16 days HH/GLI repression (*K5GLIR*) (B, 28%), or 16 days HH/GLI repression followed by 7 days Recovery (C, 25%). **D,E,F**. TYPE II, Atypical FP/Atypical TB, and percentages in Control (D,4%), HH/GLI Repressed (E,47%) and Recovery (F, 32%) mice. After 16 days of HH/GLI repression, 47% of FP have lost *lacZ*-positive cells in the epithelium but not in the stroma (E). Some FP, about 32%, exhibit a few epithelial *lacZ*-positive cells already at 7 days Recovery (F). **G,H,I**. TYPE III, Atypical FP/No TB, and percentages in Control tongue (G, 12%), HH/GLI Repressed (H, 25%) and 7 days Recovery (I, 43%). In Type III FP with no TB, and therefore no SHH, there are no *lacZ*-positive HH-responding cells in the FP epithelium. These do not recover from HH/GLI Repression but remain at a large percentage of all FPs (I, 43%). Note that after 7 days recovery from HH/GLI repression, 25% of Typical, TYPE I FP/TB have a distribution of *lacZ*-positive cells in FP epithelial walls and the stromal core (C), whereas TYPE III Atypical FP/No TB have no *lacZ*-positive cells in the epithelium (I).
(TIF)

S10 Fig Recovery of epithelial HH-responding *Gli1lacZ*+ cells in circumvallate papilla after stopping HH/GLI blockade. *Gli1 lacZ*-positive cells in circumvallate papilla (left wall) of Control and after 5 days conditional gene deletion after (5d *Gli2cKO*) mouse, and after 14 days treatment cessation from prior 16 days HH/GLI repression (16d *K5GliR*/14d Recovery). The same sections with K8 immunostaining are paired below, to show labeled taste bud cells. With conditional GLI blockade taste bud cells are lost and HH-responding cells are eliminated from the CV epithelium (5d *Gli2cKO*; and see Fig 9). After 14 days recovery from treatment to repress HH/GLI signaling, *Gli1 lacZ*-positive cells are in the epithelium surrounding taste bud cells and in stromal cells, comparable to Control. Scale bar applies to all images. Dotted lines indicate the extent of the papilla epithelium in two middle panels.
(TIF)
