# Supplementary figures and images for: Maintenance of Taste Organs Is Strictly Dependent on Epithelial Hedgehog/GLI Signaling
Source: PLoS Genet. 2016 Nov 28;12(11):e1006442. doi: 10.1371/journal.pgen.1006442 (PMC5125561; doi:10.1371/journal.pgen.1006442)

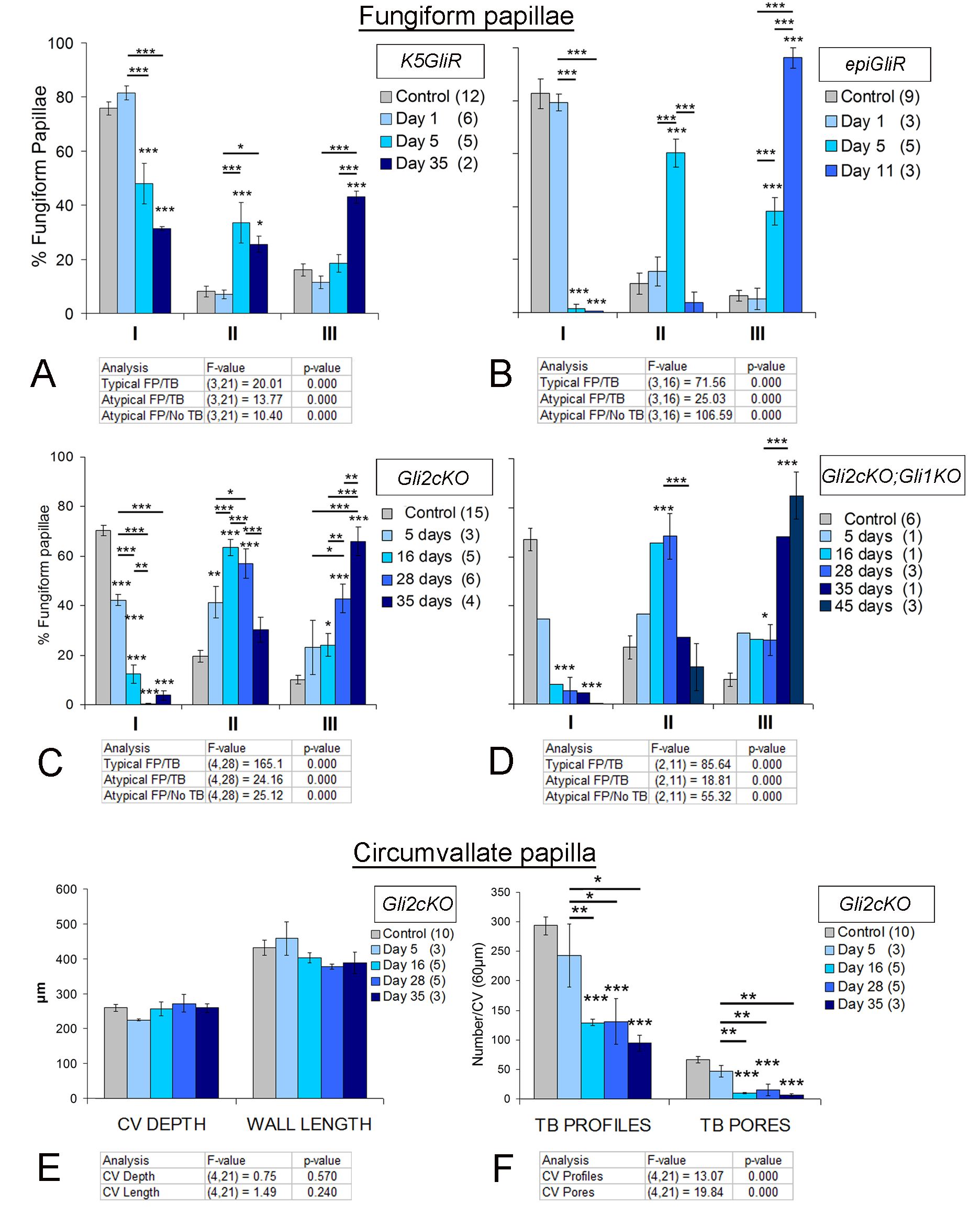

Supplement: S1 Fig — A,B,C,D. ANOVA data with posthoc comparisons for FP effects in Figs 1 and 2 after HH/GLI repression (A, K5GliR; B, epiGliR) and gene deletion (C, Gli2cKO;D, Gli2cKO;Gli1KO). E,F. ANOVA data with posthoc comparisons for effects of Gli2cKO in Fig 7 on CV structure and numbers of taste bud profiles/remnants or taste pores (E,F). Significance levels are indicated (*p<0.05; **p<0.01; ***p<0.001). (TIF) [file pgen.1006442.s001.tif]

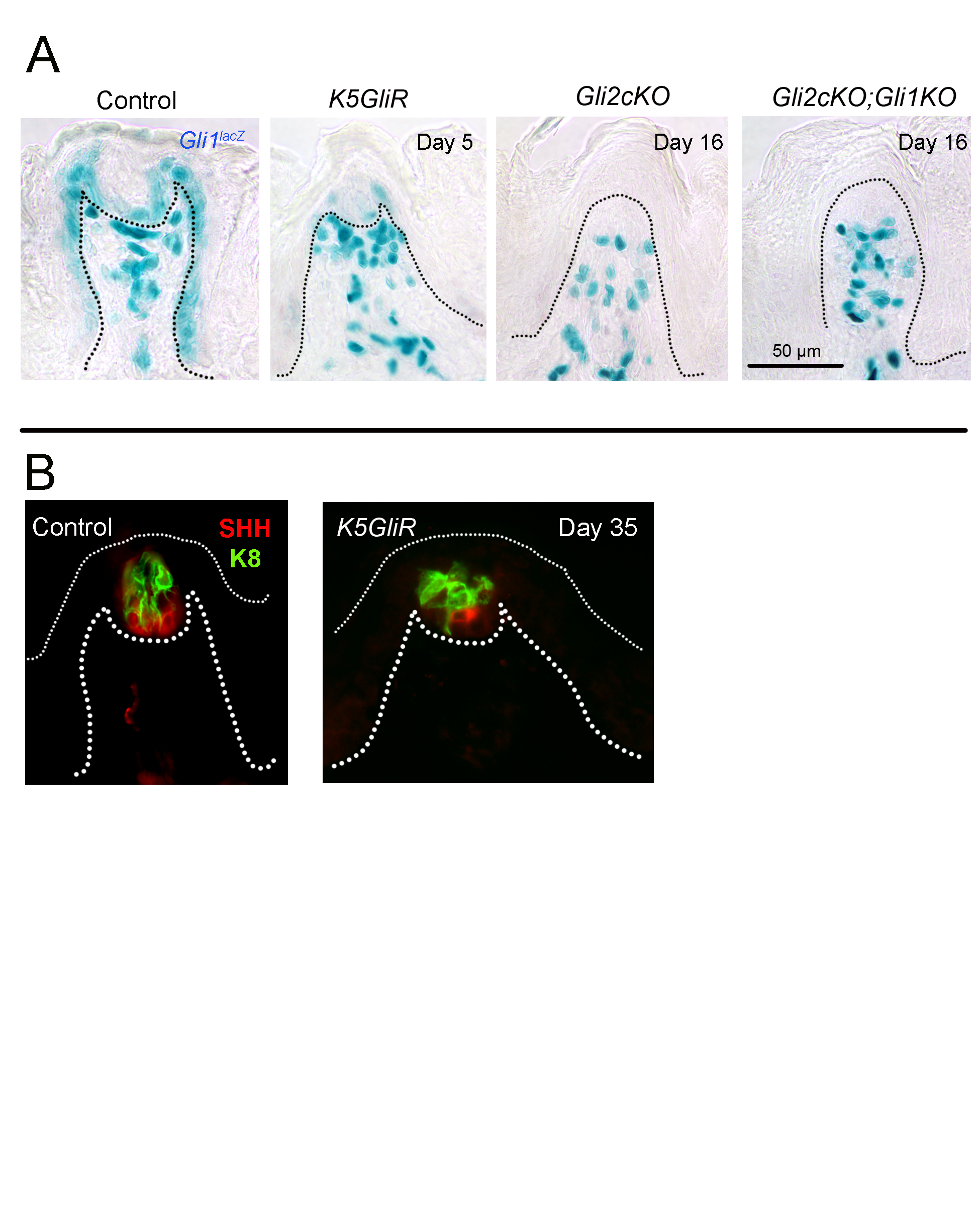

Supplement: S2 Fig — A. X-Gal staining to detect β-gal-positive, HH-responding cells in Control Gli1lacZ/+, and K5GliR, Gli2cKO, and Gli2cKO;Gli1KO mice. After transgene activation there is loss of detectable HH signaling in epithelium, with lacZ-positive cells in the FP stroma only. B. Compared to Control, very few taste bud remnants are observed after 35 days HH/GLI repression in K5GliR tongues and SHH ligand is reduced, associated with the taste cell loss. Scale bar applies to all images. (TIF) [file pgen.1006442.s002.tif]

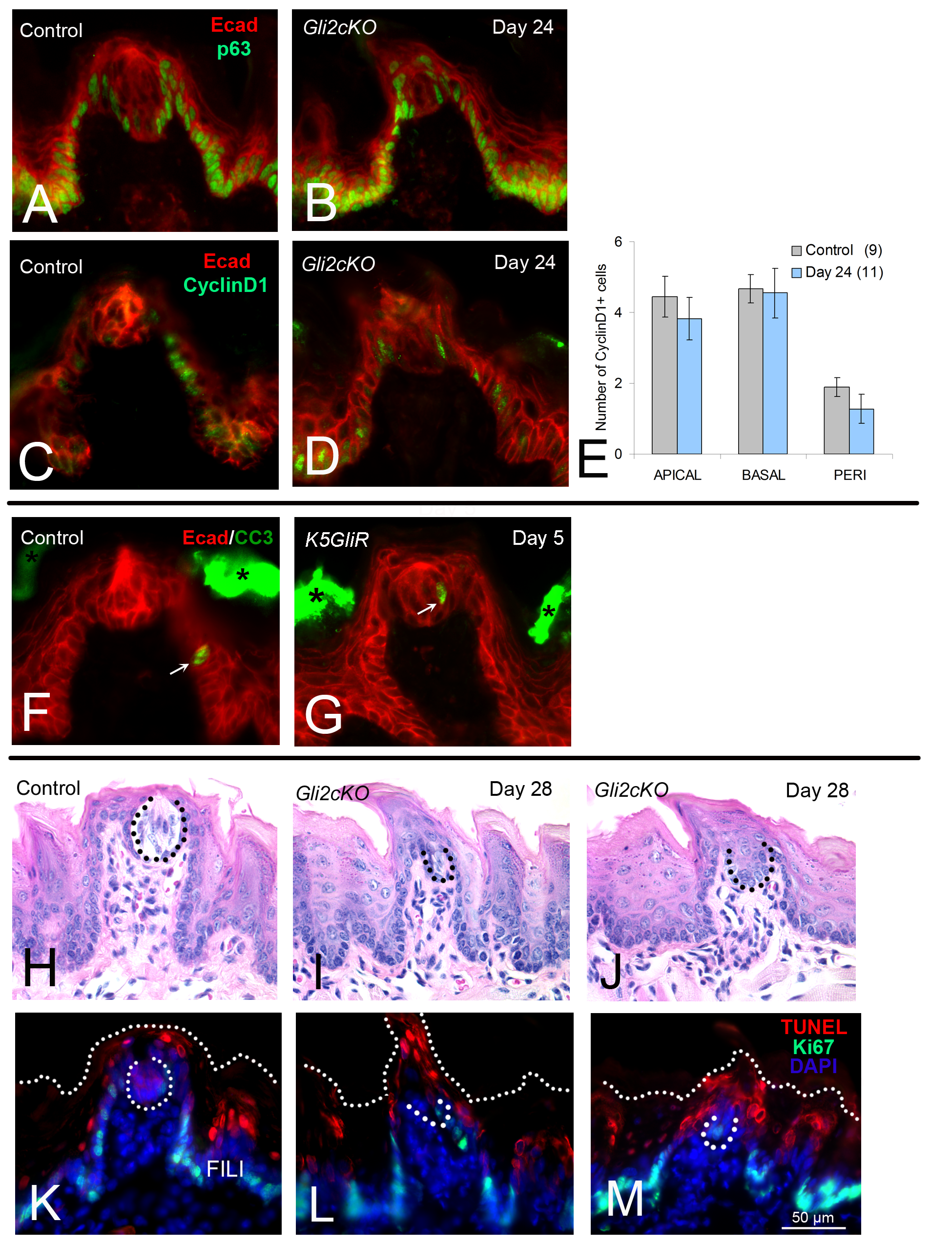

Supplement: S3 Fig — A,B, FP immunostained for p63 and E-cadherin to label the epithelium, in Control (A) and Gli2cKO tongue (B). p63-positive cells are continuous and apparently homogeneous throughout the basal epithelial cells of the tongue and papillae in control tongue and after HH signaling repression. C,D,E. Cyclin D1 immunostaining in FP in Control (C) and Gli2cKO (D) tongues and graph (E) for cell counts of Cyclin D1-positve cells in APICAL, BASAL and PERIGEMMAL regions of the FP (compare to Fig 4H for Ki67-positive cells). Cyclin D1-positive cells tended to decrease in APICAL and PERIGEMMAL regions in FP from tongues after HH suppression compared to Control, but there were no significant differences. Numbers in parentheses indicate number of FP analyzed. F,G. Cleaved Caspase 3-positive cells (CC3, arrows) with Ecadherin (Ecad) immunostaining indicates infrequent CC3 label in Control and K5GliR FP. H,I,J. H& E sections to illustrate phenotype for Control (H), and two examples of disrupted FP and taste buds after gene deletion (Gli2cKO) for 28 days (I,J). The taste bud or taste bud remnant is circled in all images. K,L,M. TUNEL staining in Control (K) and two examples for Gli2cKO tongues to label dying cells (L,M), with Ki67 immunoreactions for proliferating cells. Taste buds or remnants are circled in each image. In Control FP (K) TUNEL-positive cells are in suprabasal regions of the epithelium and FP apex. In filiform papillae (FILI) there are accumulated TUNEL-positive cells in suprabasal apical regions. In two examples from Gli2cKO tongues of disrupted FP and taste bud cell remnants (L,M) compared to Control the extent of TUNEL stained cells at the extreme papilla apex, which becomes conical and heavily keratinized, has apparently increased somewhat. Dotted lines demarcate the border of the superficial epithelial cells that are beneath the keratinized surface squames. Scale bar in M applies to all images. (TIF) [file pgen.1006442.s003.tif]

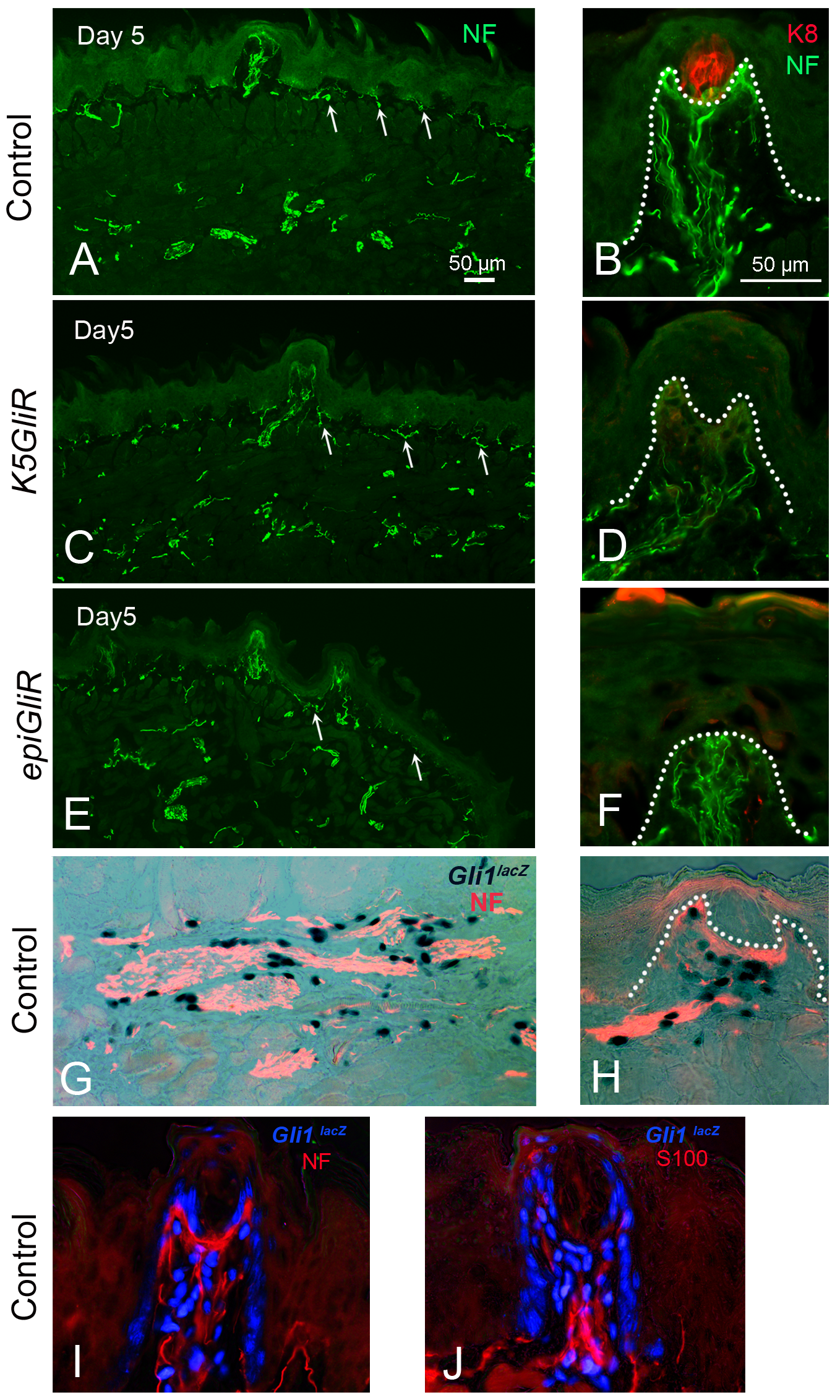

Supplement: S4 Fig — A,B; C,D; E,F. Anterior tongue sections and sample FP from Control (A,B), K5GliR (C,D) and epiGliR (E,F) tongues after 5 days. K8 labels taste bud cells and neurofilament (NF) labels lingual innervation. With HH/GLI repression, nerves are not lost or redirected but track under the epithelium and turn to densely innervate FPs (A,C,E, arrows). Even FPs without taste bud cell remnants are innervated (D,F). G,H. Gli1lacZ-positive cells and NF immunostaining in Control tongue illustrate that HH-responding cells are in direct association with, or lining, nerves in the tongue body (G) and going into the FP (H). I,J. NF labeled fibers (I) and S100-positive Schwann cells (J) are in direct association with Gli1 lacZ-positive, HH-responding cells in FP. (TIF) [file pgen.1006442.s004.tif]

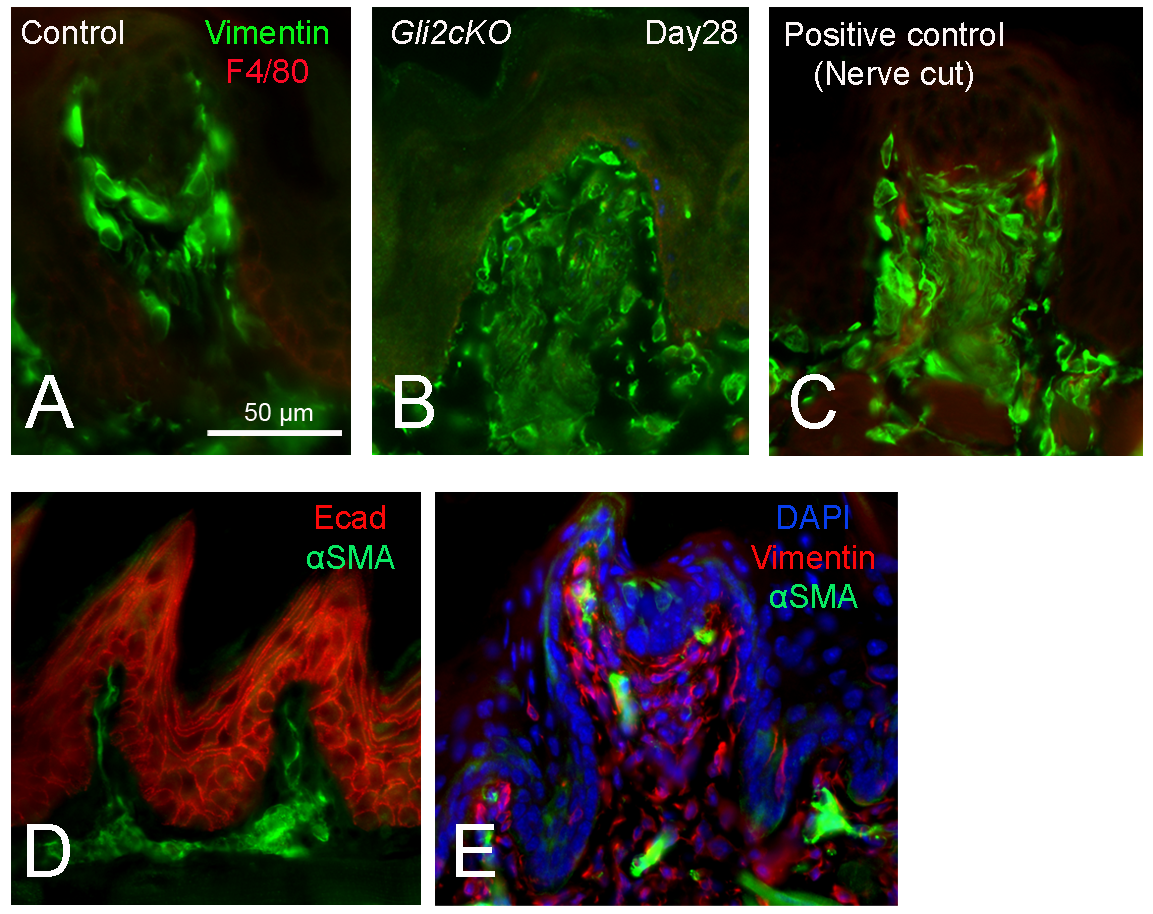

Supplement: S5 Fig — F4/80 immunostaining for macrophages, with vimentin reactions, in Control (A) and conditional Gli2 deletion (B) tongues, demonstrate absence of macrophage invasion in FP with HH suppression. As a positive control for the macrophage labeling we used tongues with lingual nerve cuts, a procedure known to increase macrophage invasion (C). D,E. To label myofibroblasts we used immunostaining for smooth muscle actin (αSMA) and identified cells within the stroma core of filiform (D) and fungiform (E) papillae. Ecad and DAPI demarcated the epithelium. (TIF) [file pgen.1006442.s005.tif]

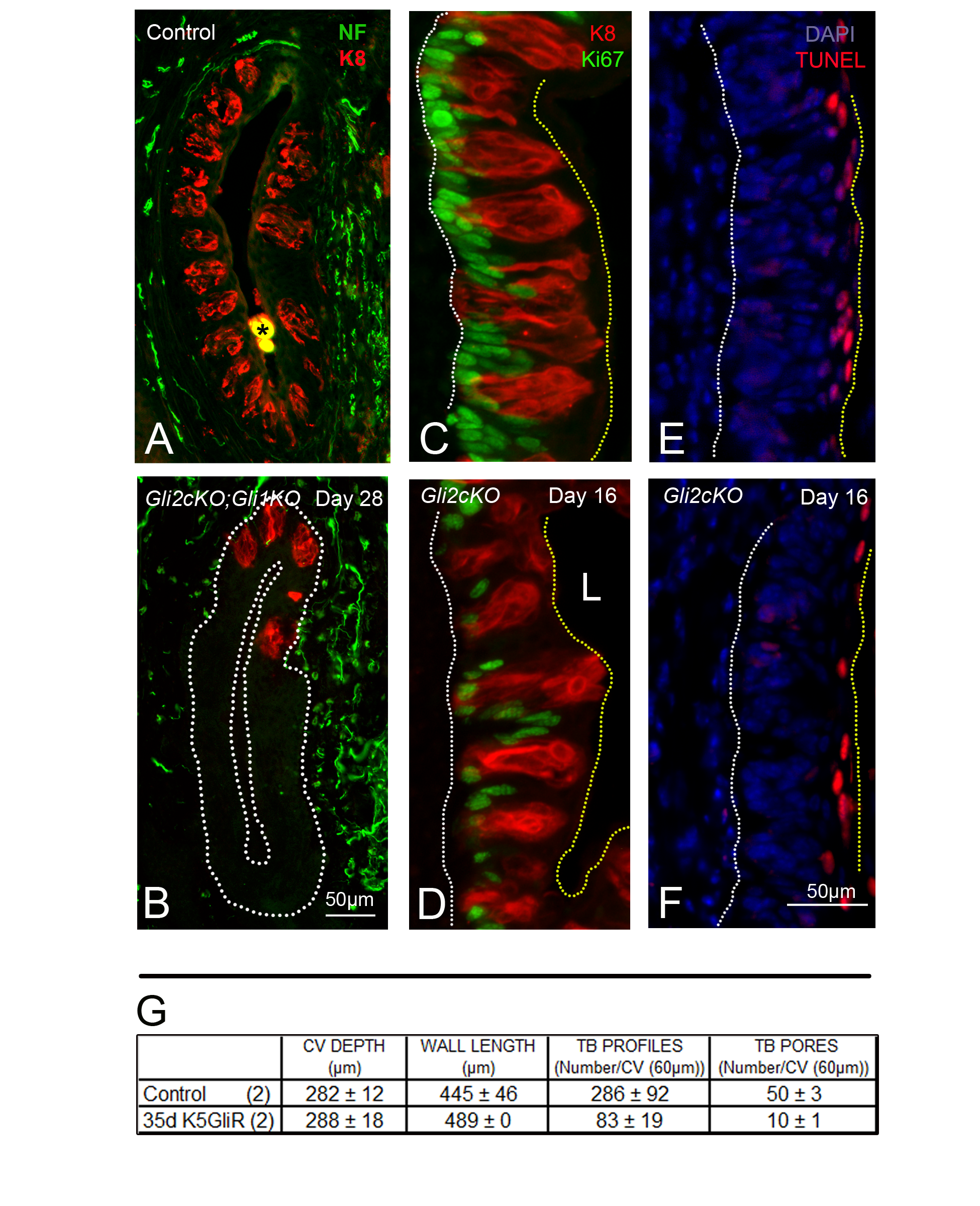

Supplement: S6 Fig — A,B. Control circumvallate papilla (A), with K8 immunostaining to label taste bud cells, has extensive innervation around papilla walls as seen with NF immunostaining. (Asterisk in A indicates nonspecific fluorescence.) With conditional gene deletion after 28 days in Gli2cKO;Gli1KO tongue, nerves are retained around the circumvallate papilla walls (B). Taste bud cells are much reduced. C,D. Control papilla (C), with K8 to label taste bud cells, and Ki67 immunostaining to label proliferating cells illustrates Ki67-positive cells in basal epithelium of the papilla. In Gli2cKO tongue (D), the taste bud cells and Ki67-positive cells are much reduced. E,F. In Control (E) and Gli2cKO (F) papilla, TUNEL staining labels cells seen in suprabasal regions near the luminal surface of the papilla. The extent of TUNEL-positive staining is not noticeably different in Control and Gli2cKO CV. Dotted lines indicate the extent of the papilla epithelium. In panel D, an ‘L’ denotes the luminal side of the papilla epithelium that applies to C,D,E,F. G. Data Table for results comparing CV in Control tongues with K5GliR tongues after 35 days HH suppression. Whereas CV size is not different, taste pores are reduced in K5GliR CV (t = 14.7, p = 0.005). Scale bar in B applies to A and B; scale bar in F applies to C,D,E,F. (TIF) [file pgen.1006442.s006.tif]

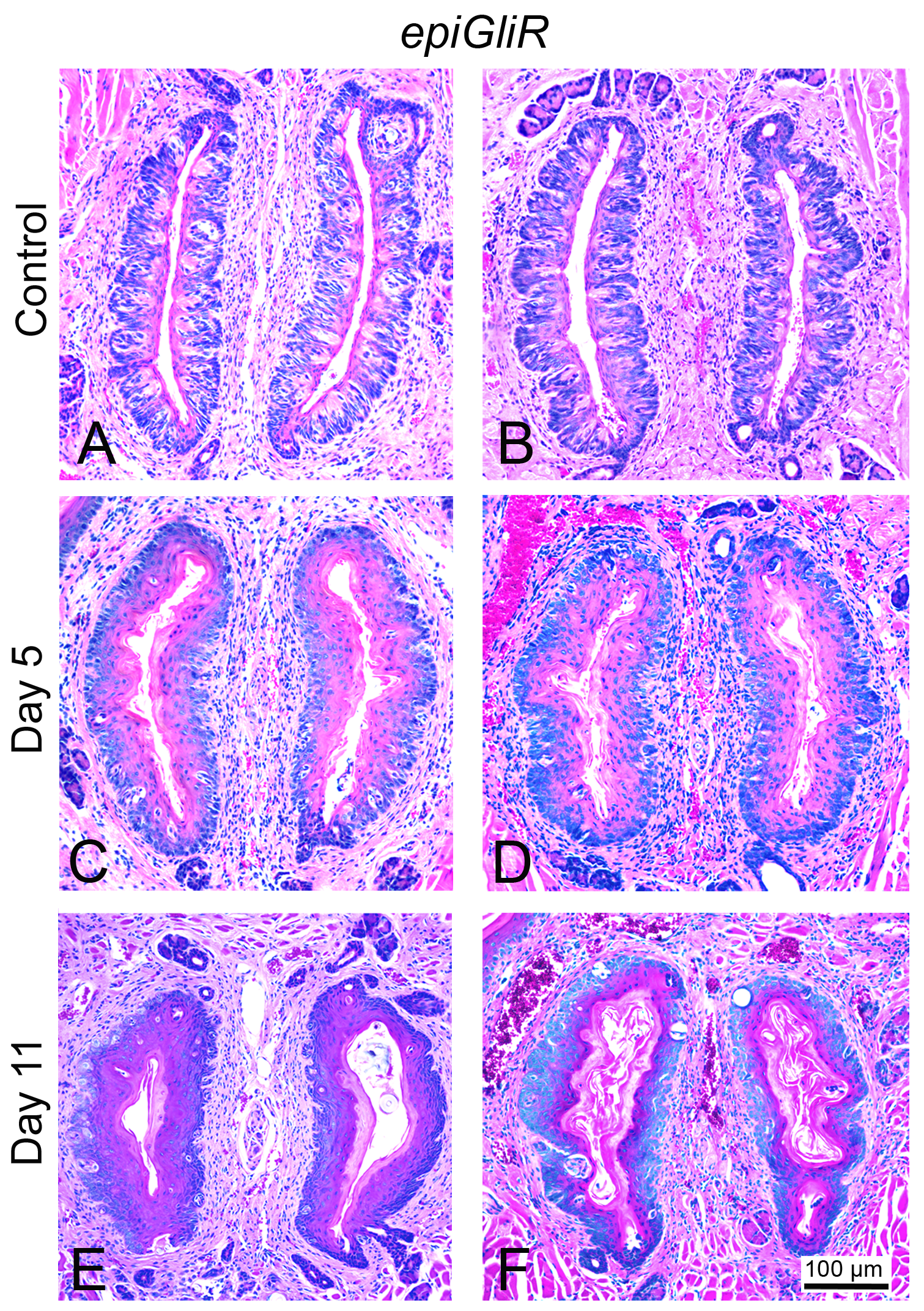

Supplement: S7 Fig — A,B. H&E sections through the mid-region of circumvallate papillae from two Control tongues, to illustrate complete papilla structure and epithelium replete with taste buds. C,D. Mid-region of circumvallate papillae from two tongues with HH/GLI repression, at 5 days after epiGliR transgene activation. E,F. Mid-region of circumvallate papillae from two tongues, at 11 days after epiGliR transgene activation. Taste buds are not observed within the papilla epithelium. Scale bar in F applies to all images. (TIF) [file pgen.1006442.s007.tif]

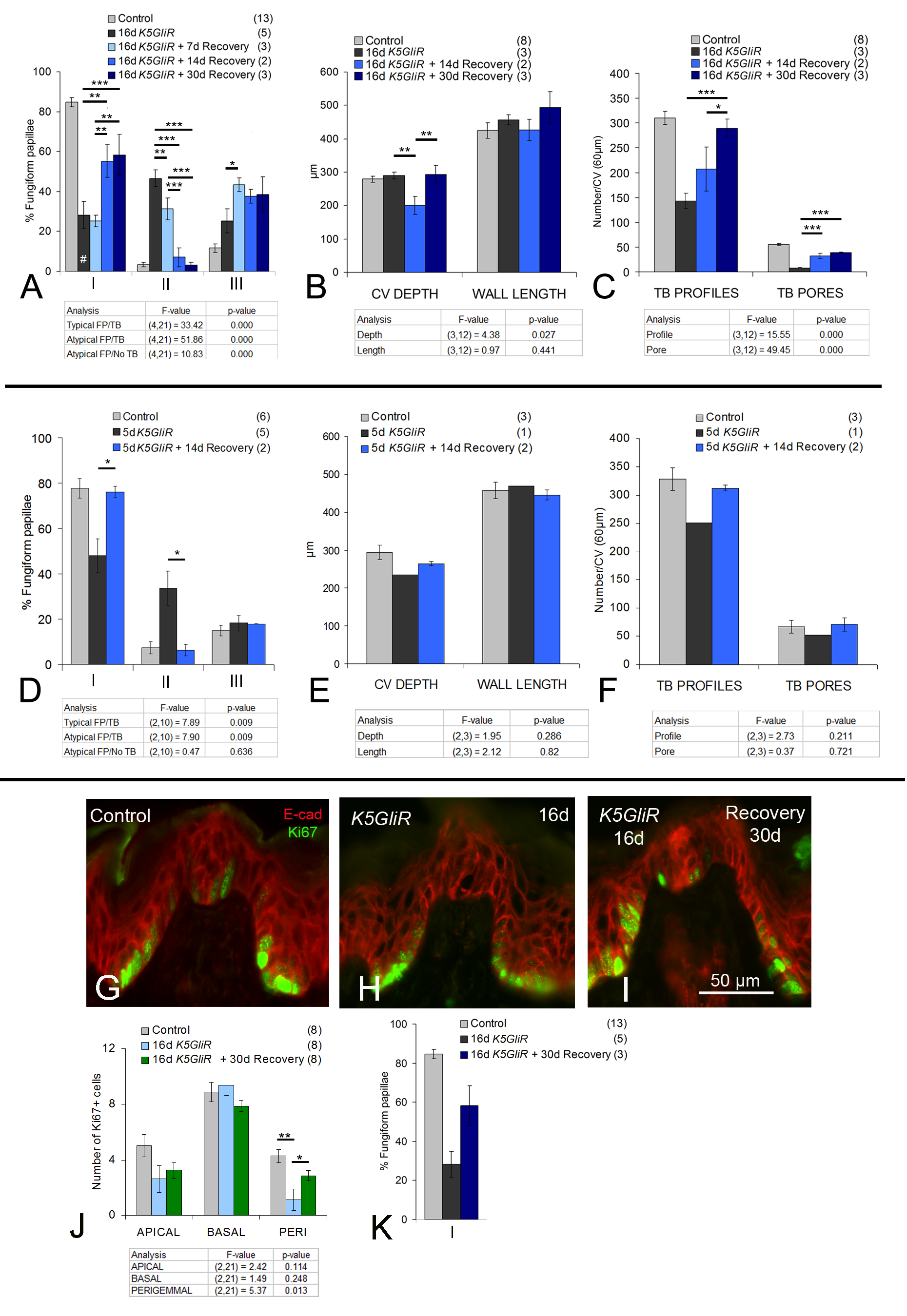

Supplement: S8 Fig — A,B,C. ANOVA data for effects of recovery at 7 to 30 days after HH/GLI repression for 16 days in FP (A) and CV (B,C), presented in Fig 9. D,E,F. Effects for 5 days HH/GLI repression and subsequent 14 days of recovery in FP (D) and CV (E,F). After a shorter repression duration of 5 days, compared to A,B,C, the recovery to TYPE I Typical FB/TB is essentially complete (D). CV depth and wall length are not different in any groups (E). Recovery to full numbers of TB profiles and pores is seen after 14 days (F). Numbers of tongues at each time point are indicated in parentheses in graph legends. G,H,I,J,K. Ki67-positive, proliferating cells in Control FP (G), K5GliR FP after 16 days HH suppression (H) and K5GliR FP after 30 days recovery from 16 days of HH suppression (I). Cell counts in APICAL, BASAL and PERIGEMMAL regions of the FP are graphed (J). Numbers of FP at each time point are indicated in parentheses in graph legend. The extent of recovery of Ki67-positive cells is directly comparable to that of Type I Typical FP/TB (K; data are extracted from Fig 9D, and S8A Fig). (TIF) [file pgen.1006442.s008.tif]

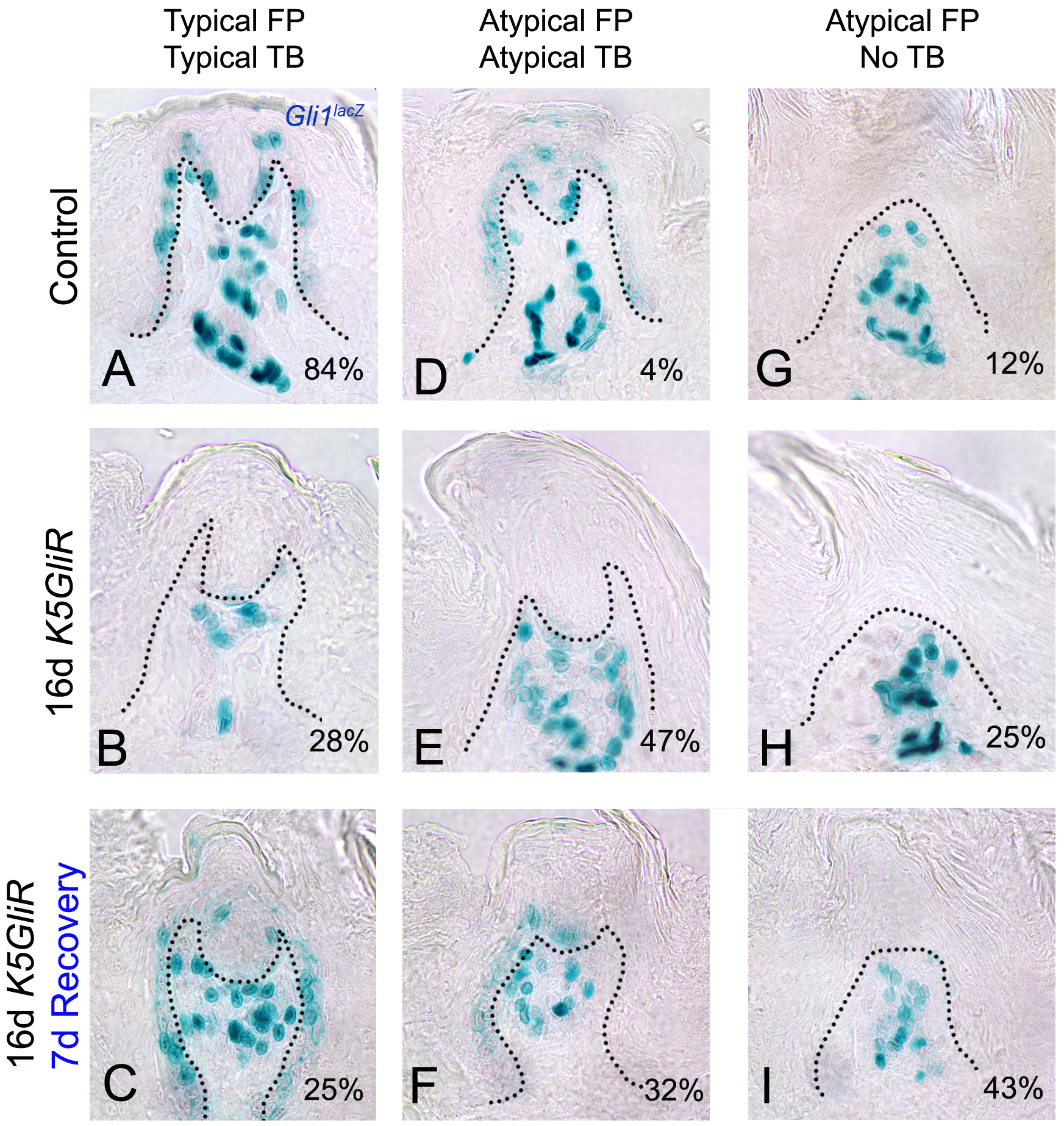

Supplement: S9 Fig — The distribution of HH- responding cells, Gli1lacZ positive, after Recovery matches presence of Typical or Atypical taste buds in specific fungiform papillae. A,B,C. Examples with percentages of representation for TYPE I, Typical FP/TB, in tongues of Control mice (A, 84%), after 16 days HH/GLI repression (K5GLIR) (B, 28%), or 16 days HH/GLI repression followed by 7 days Recovery (C, 25%). D,E,F. TYPE II, Atypical FP/Atypical TB, and percentages in Control (D,4%), HH/GLI Repressed (E,47%) and Recovery (F, 32%) mice. After 16 days of HH/GLI repression, 47% of FP have lost lacZ-positive cells in the epithelium but not in the stroma (E). Some FP, about 32%, exhibit a few epithelial lacZ-positive cells already at 7 days Recovery (F). G,H,I. TYPE III, Atypical FP/No TB, and percentages in Control tongue (G, 12%), HH/GLI Repressed (H, 25%) and 7 days Recovery (I, 43%). In Type III FP with no TB, and therefore no SHH, there are no lacZ-positive HH-responding cells in the FP epithelium. These do not recover from HH/GLI Repression but remain at a large percentage of all FPs (I, 43%). Note that after 7 days recovery from HH/GLI repression, 25% of Typical, TYPE I FP/TB have a distribution of lacZ-positive cells in FP epithelial walls and the stromal core (C), whereas TYPE III Atypical FP/No TB have no lacZ-positive cells in the epithelium (I). (TIF) [file pgen.1006442.s009.tif]

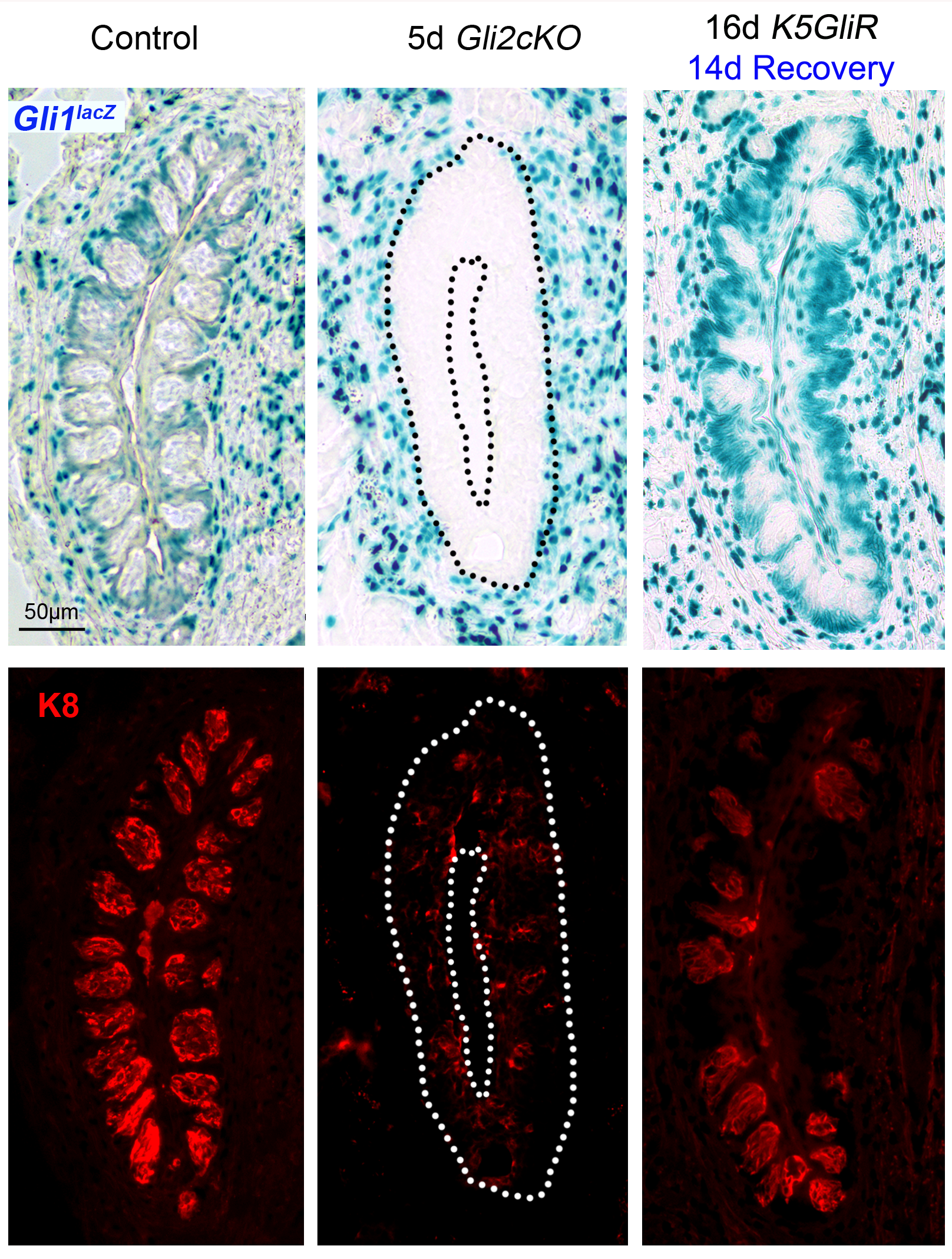

Supplement: S10 Fig — Gli1 lacZ-positive cells in circumvallate papilla (left wall) of Control and after 5 days conditional gene deletion after (5d Gli2cKO) mouse, and after 14 days treatment cessation from prior 16 days HH/GLI repression (16d K5GliR/14d Recovery). The same sections with K8 immunostaining are paired below, to show labeled taste bud cells. With conditional GLI blockade taste bud cells are lost and HH-responding cells are eliminated from the CV epithelium (5d Gli2cKO; and see Fig 9). After 14 days recovery from treatment to repress HH/GLI signaling, Gli1 lacZ-positive cells are in the epithelium surrounding taste bud cells and in stromal cells, comparable to Control. Scale bar applies to all images. Dotted lines indicate the extent of the papilla epithelium in two middle panels. (TIF) [file pgen.1006442.s010.tif]
